# Supplementary material for: The influencing factors of biomedical R&D cooperation in three major urban agglomerations of China based on cooperative patents
Source: PLoS One. 2023 Jan 4;18(1):e0278942. doi: 10.1371/journal.pone.0278942 (PMC9812333; doi:10.1371/journal.pone.0278942)
Supplement: S1 Data — (ZIP) [file pone.0278942.s001.zip › Original Files/2008-2010the Pearl River Delta Urban Agglomeration.pdf]

City pair	High-speed rail	Tier 1 cities	Different provinces	Capital city	Bay Area Center	Frequency
Guangzhou——Dongguan	0	1	0	1	1	3
Zhongshan——Zhaoqing	0	0	0	0	0	1
Guangzhou——Shenzhen	1	1	0	1	1	1
Guangzhou——Foshan	0	1	0	1	1	8
Guangzhou——Guangzhou						60
Shenzhen——Shenzhen						22
Zhongshan——Zhongshan						3
Zhuhai——Zhuhai						9
