# Supplementary material for: The influencing factors of biomedical R&D cooperation in three major urban agglomerations of China based on cooperative patents
Source: PLoS One. 2023 Jan 4;18(1):e0278942. doi: 10.1371/journal.pone.0278942 (PMC9812333; doi:10.1371/journal.pone.0278942)
Supplement: S1 Data — (ZIP) [file pone.0278942.s001.zip › Original Files/2008-2010Yangtze River Delta Urban Agglomeration.pdf]

City pair	High-speed rail	Tier 1 cities	Different provinces	Capital city	Bay Area Center	Frequency
Nanjing——Nantong	0	0	0	1	0	1
Shanghai——Jiaxing	1	1	1	0	1	8
Shanghai——Jinhua	0	1	1	0	1	7
Shanghai——Taizhou1	0	1	1	0	1	8
Hefei——Anqing	0	0	0	1	0	1
Nanjing——Taizhou2	0	0	0	1	0	28
Nantong——Zhenjiang	0	0	0	0	0	1
Shanghai——Hangzhou	1	1	1	1	1	6
Ningbo——Hefei	0	0	1	1	0	1
Shanghai——Nantong	0	1	1	0	1	3
Shanghai——Nanjing	1	1	1	1	1	18
Shanghai——Wuxi	1	1	1	0	1	5
Suzhou——Nanjing	1	0	0	1	0	1
Hefei——Chizhou	0	0	0	1	0	1
Ningbo——Taizhou1	1	0	0	0	0	1
Shanghai——Hefei	1	1	1	1	1	8
Hangzhou——Nanjing	1	0	1	1	0	6

Hangzhou——Nantong	0	0	1	1	0	1
Hangzhou——Taizhou1	0	0	0	1	0	6
Nanjing——Maanshan	0	0	1	1	0	32
Changzhou——Yancheng	0	0	0	0	0	14
Hangzhou——Jinhua	0	0	0	1	0	3
Shanghai——Shaoxing	0	1	1	0	1	6
Nanjing——Wuxi	1	0	0	1	0	5
Nanjing——Jinhua	0	0	1	1	0	5
Shanghai——Huzhou	0	1	1	0	1	2
Shanghai——Taizhou2	0	1	1	0	1	4
Shanghai——Suzhou	1	1	1	0	1	29
Suzhou——Wuxi	1	0	0	0	0	2
Hangzhou——Zhoushan	0	0	0	1	0	1
Suzhou——Nantong	0	0	0	0	0	1
Zhenjiang——Taizhou2	0	0	0	0	0	1
Nanjing——Zhenjiang	1	0	0	1	0	2
Shanghai——Ningbo	0	1	1	0	1	1
Nanjing——Jiaxing	0	0	1	1	0	2

Wuxi——Nantong	0	0	0	0	0	1
Hangzhou——Huzhou	0	0	0	1	0	1
Hangzhou——Shaoxing	0	0	0	1	0	4
Hangzhou——Ningbo	0	0	0	1	0	1
Shanghai——Changzhou	1	1	1	0	1	6
Hangzhou——Jiaxing	1	0	0	1	0	1
Shanghai——Yangzhou	0	1	1	0	1	2
Nanjing——Changzhou	1	0	0	1	0	4
Yancheng——Zhenjiang	0	0	0	0	0	1
Shanghai——Yancheng	0	1	1	0	1	2
Shanghai——Zhenjiang	1	1	1	0	1	2
Hangzhou——Wuxi	0	0	1	1	0	1
Shanghai——Shanghai						378
Yangzhou——Yangzhou						3
Hangzhou——Hangzhou						54
Jiaxing——Jiaxing						5
Wuxi——Wuxi						3

Nanjing——Nanjing	118
Zhoushan——Zhoushan	1
Hefei——Hefe	12
Suzhou——Suzhou	6
Shaoxing——Shaoxing	4
Changzhou——Changzhou	12
Yancheng——Yancheng	2
Taizhou2——Taizhou2	3
Jinhua——Jinhua	2
Zhenjiang——Zhenjiang	3
Nantong——Nantong	1
