# Supplementary material for: The influencing factors of biomedical R&D cooperation in three major urban agglomerations of China based on cooperative patents
Source: PLoS One. 2023 Jan 4;18(1):e0278942. doi: 10.1371/journal.pone.0278942 (PMC9812333; doi:10.1371/journal.pone.0278942)
Supplement: S1 Data — (ZIP) [file pone.0278942.s001.zip › Original Files/2011-2013Beijing-Tianjin-Hebei Urban Agglomeration.pdf]

City pair	High-speed rail	Tier 1 cities	Different provinces	Capital city	Bay Area Center	Frequency
Beijing——Shijiazhuang	1	1	1	1	1	8
Beijing——Tianjin	1	1	1	0	1	20
Beijing——Chengdei	0	1	1	0	1	2
Shijiangzhuang——Qinhuangdao	0	0	0	1	0	2
Beijing——Baoding	1	1	1	0	1	1
Beijing——Cangzhou	1	1	1	0	1	1
Beijing——Qinhuangdao	1	1	1	0	1	1
Beijing——Handan	1	1	1	0	1	1
Beijing——Beijing						392
Tianjin——Tianjin						99
Shijiangzhuang——Shijiangzhuang						11
