# Supplementary material for: The influencing factors of biomedical R&D cooperation in three major urban agglomerations of China based on cooperative patents
Source: PLoS One. 2023 Jan 4;18(1):e0278942. doi: 10.1371/journal.pone.0278942 (PMC9812333; doi:10.1371/journal.pone.0278942)
Supplement: S1 Data — (ZIP) [file pone.0278942.s001.zip › Original Files/2011-2013Yangtze River Delta Urban Agglomeration.pdf]

City pair	High-speed rail	Tier 1 cities	Different provinces	Capital city	Bay Area Center	Frequency
Zhenjiang——Taizhou2	0	0	0	0	0	2
Nanjing——Zhenjiang	1	0	0	1	0	26
Hangzhou——Nantong	0	1	1	1	0	4
Shanghai——Hefei	1	1	1	1	1	3
Hangzhou——Nanjing	1	1	1	1	0	4
Shanghai——Wuxi	1	1	1	0	1	18
Shanghai——Yangzhou	0	1	1	0	1	1
Shanghai——Nanjing	1	1	1	1	1	24
Shanghai——Suzhou	1	1	1	0	1	41
Suzhou——Nanjing	1	1	0	1	0	22
Shanghai——Shaoxing	1	1	1	0	1	16
Shanghai——Taizhou1	1	1	1	0	1	1
Shanghai——Hangzhou	1	1	1	1	1	18
Shanghai——Zhenjiang	1	1	1	0	1	11
Shanghai——Changzhou	1	1	1	0	1	25

Hangzhou——Changzhou	1	1	1	1	0	2
Nanjing——Hefei	1	1	1	1	0	9
Nanjing——Maanshan	0	1	1	1	0	3
Nanjing——Taizhou2	0	1	0	1	0	10
Shanghai——Jinhua	0	1	1	0	1	1
Nanjing——Changzhou	1	1	0	1	0	5
Hangzhou——Shaoxing	1	1	0	1	0	16
Shanghai——Maanshan	0	1	1	0	1	3
Suzhou——Hefei	1	0	1	1	0	1
Hangzhou——Ningbo	1	1	0	1	0	4
Hangzhou——Jinhua	0	1	0	1	0	2
Changzhou——Yancheng	0	0	0	0	0	10
Nanjing——Nantong	0	1	0	1	0	2
Shanghai——Yancheng	0	1	1	0	1	3
Nanjing——Yancheng	0	1	0	1	0	7
Hangzhou——Taizhou1	1	1	0	1	0	1
Shanghai——Ningbo	1	1	1	0	1	1

Shanghai——Nantong	0	1	1	0	1	5
Hefei——Chizhou	0	0	0	1	0	2
Nanjing——Wuxi	1	1	0	1	0	4
Suzhou——Wuxi	1	1	0	0	0	1
Shanghai——Anqing	0	1	1	0	1	4
Nanjing——Yangzhou	0	1	0	1	0	1
Wuxi——Changzhou	1	1	0	0	0	2
Suzhou——Zhenjiang	1	0	0	0	0	7
Hefei——Tongling	0	0	0	1	0	2
Nanjing——Huzhou	1	1	1	1	0	1
Changzhou——Zhenjiang	1	0	0	0	0	1
Nanjing——Jinhua	0	1	1	1	0	1
Hefei——Xuancheng	0	0	0	1	0	1
Hangzhou——Huzhou	1	1	0	1	0	1
Wuxi——Zhenjiang	1	1	0	0	0	1
Yangzhou——Nantong	0	0	0	0	0	2
Wuxi——Nantong	0	1	0	0	0	1
Shanghai——Shanghai						482

Hangzhou——Hangzhou	58
Shaoxing——Shaoxing	33
Nanjing——Nanjing	119
Taizhou2——Taizhou2	3
Changzhou——Changzhou	26
Suzhou——Suzhou	26
Yancheng——Yancheng	4
Jinhua——Jinhua	7
Wuxi——Wuxi	8
Taizhou1——Taizhou1	2
Hefei——Hefei	16
Zhenjiang——Zhenjiang	4
Yangzhou——Yangzhou	1
Huzhou——Huzhou	1
Chuzhou——Chuzhou	1
Zhoushan——Zhoushan	1
Nantong——Nantong	10
Ningbo——Ningbo	1
