# Supplementary material for: The influencing factors of biomedical R&D cooperation in three major urban agglomerations of China based on cooperative patents
Source: PLoS One. 2023 Jan 4;18(1):e0278942. doi: 10.1371/journal.pone.0278942 (PMC9812333; doi:10.1371/journal.pone.0278942)
Supplement: S1 Data — (ZIP) [file pone.0278942.s001.zip › Original Files/2014-2016the Pearl River Delta Urban Agglomeration.pdf]

City pair	High-speed rail	Tier 1 cities	Different provinces	Capital city	Bay Area Center	Frequency
Guangzhou——Dongguan	1	1	0	1	1	61
Guangzhou——Foshan	0	1	0	1	1	21
Guangzhou——Zhuhai	1	1	0	1	1	17
Guangzhou——Shenzhen	1	1	0	1	1	27
Guangzhou——Zhongshan	1	1	0	1	1	4
Shenzhen——Dongguan	1	1	0	0	0	8
Shenzhen——Foshan	0	1	0	0	0	4
Guangzhou——Jiangmen	0	1	0	1	1	3
Shenzhen——Huizhou	1	1	0	0	0	1
Shenzhen——Zhuhai	1	1	0	0	0	3
Zhongshan——Foshan	0	0	0	0	0	1
Guangzhou——Huizhou	1	1	0	1	1	1
Zhuhai——Zhongshan	1	0	0	0	0	1
Guangzhou——Zhaoqing	1	1	0	1	1	12
Dongguan——Dongguan						27

Guangzhou——Guangzhou	299
Shenzhen——Shenzhen	659
Foshan——Foshan	43
Zhuhai——Zhuhai	10
Huizhou——Huizhou	10
Jiangmen——Jiangmen	2
Zhongshan——Zhongshan	4
Shenzhen——Zhuhai	3
