# Supplementary material for: The influencing factors of biomedical R&D cooperation in three major urban agglomerations of China based on cooperative patents
Source: PLoS One. 2023 Jan 4;18(1):e0278942. doi: 10.1371/journal.pone.0278942 (PMC9812333; doi:10.1371/journal.pone.0278942)
Supplement: S1 Data — (ZIP) [file pone.0278942.s001.zip › Original Files/2014-2016Yangtze River Delta Urban Agglomeration.pdf]

City pair	High-speed rail	Tier 1 cities	Different provinces	Capital city	Bay Area Center	Frequency
Nanjing——Taizhou2	1	1	0	1	0	6
Shangha——Anqing	1	1	1	0	1	1
Nanjing——Zhenjiang	1	1	0	1	0	49
Nanjing——Suzhou	1	1	0	1	0	63
Suzhou——Zhenjiang	1	1	0	0	0	28
Nanjing——Yangzhou	1	1	0	1	0	7
Shangha——Hangzhou	1	1	1	1	1	48
Nanjing——Hefei	1	1	1	1	0	3
Shangha——Shaoxing	1	1	1	0	1	16
Shangha——Suzhou	1	1	1	0	1	41
Hangzhou——Jinhua	1	1	0	1	0	5
Shangha——Wuxi	1	1	1	0	1	17
Nanjing——Changzhou	1	1	0	1	0	4
Nanjing——Yancheng	0	1	0	1	0	5
Shangha——Zhenjiang	1	1	1	0	1	4

Hangzhou——Nanjing	1	1	1	1	0	3
Shangha——Nanjing	1	1	1	1	1	12
Shangha——Maanshan	1	1	1	0	1	6
Nanjing——Wuxi	1	1	0	1	0	5
Hangzhou——Jiaxing	1	1	0	1	0	10
Shangha——Ningbo	1	1	1	0	1	11
Shangha——Changzhou	1	1	1	0	1	12
Shangha——Taizhou2	0	1	1	0	1	21
Hangzhou——Ningbo	1	1	0	1	0	5
Suzhou——Wuxi	1	1	0	0	0	5
Hefei——Anqing	1	0	0	1	0	4
Wuxi———Changzhou	1	1	0	0	0	2
Hangzhou——Taizhou1	1	1	0	1	0	4
Shangha——Xuancheng	0	1	1	0	1	1
Hangzhou——Shaoxing	1	1	0	1	0	5
Nanjing——Taizhou1	1	1	1	1	0	1
Hangzhou——Changzhou	1	1	1	1	0	2
Shangha——Nantong	0	1	1	0	1	12

Shangha——Yangzhou	0	1	1	0	1	10
Nanjing——Jiaxing	1	1	1	1	0	2
Wuxi——Nantong	0	1	0	0	0	2
Nantong——Yancheng	0	0	0	0	0	1
Wuxi——Taizhou2	0	1	0	0	0	1
Hefei——Changzhou	1	0	1	1	0	3
Nanjing——Huzhou	1	1	1	1	0	1
Hangzhou——Hefei	1	1	1	1	0	1
Hefei——Yangzhou	0	0	1	1	0	2
Nanjing——Ningbo	1	1	1	1	0	2
Suzhou——Changzhou	1	1	0	0	0	1
Hangzhou——Zhoushan	0	1	0	1	0	1
Shangha——Hefei	1	1	1	1	1	1
Ningbo——Shaoxing	1	1	0	0	0	2
Yangzhou——Taizhou2	1	0	0	0	0	1
Shangha——Shangha						516
Nanjing——Nanjing						174
Hangzhou——Hangzhou						94

Suzhou——Suzhou	52
Changzhou——Changzhou	18
Shaoxing——Shaoxing	60
Ningbo——Ningbo	5
Hefei——Hefei	20
Zhoushan——Zhoushan	6
Wuxi——Wuxi	6
Nantong——Nantong	8
Zhenjiang——Zhenjiang	3
Yancheng——Yancheng	3
Yangzhou——Yangzhou	3
Taizhou1——Taizhou1	4
Jiaxing——Jiaxing	1
Wuhu——Wuhu	4
Taizhou2——Taizhou2	6
Jinhua——Jinhua	4
