# Supplementary material for: The influencing factors of biomedical R&D cooperation in three major urban agglomerations of China based on cooperative patents
Source: PLoS One. 2023 Jan 4;18(1):e0278942. doi: 10.1371/journal.pone.0278942 (PMC9812333; doi:10.1371/journal.pone.0278942)
Supplement: S1 Data — (ZIP) [file pone.0278942.s001.zip › Original Files/GDP and number of companies in urban agglomeration.pdf]

	GDP（100 million yuan）			Number of compani	
	2008-2010	2011-2013	2014-2016	2008-2010	2011-2013
Shanghai	15427.43	20398.52	25623.27	17794	9838
Nanjing	4391.84	7142.43	9681.51	3210	2628
Wuxi	5081.88	7406.18	8644.53	6925	5296
Changzhou	2610.38	4000.29	5316.29	5940	3917
Suzhou	8015.73	11899.55	14580.02	12405	10374
Nantong	2977.2	4596.3	6189.76	6940	4986
Yancheng	1979.35	3127.29	4208.07	3433	2775
Yangzhou	1910.59	2961.17	4054.71	3551	2630
Zhenjiang	1717.18	2638.98	3529.59	2897	2466
Taizhou2	1718.64	2729.75	3720.19	3475	2550
Hangzhou	5276.33	7721.53	10190.03	10103	6026
Ningbo	4447.21	6590.11	8100.13	12216	6862
Jiaxing	2011.18	2905.11	3577.51	6952	4339
Huzhou	1149.37	1662.5	2108.21	3333	2438
Shaoxing	2463.54	3651.11	4506.96	5341	3729
Zhoushan	555.94	852.26	1116.44	615	385
Jinhua	1852.61	2709.21	3431.83	5471	3362
Taizhou1	2139.06	2953.17	3562.79	6593	3383
Hefei	2242.86	4161.65	5705.07	1837	2158
Wuhu	993.04	1877.63	2488.77	1546	1765
Maanshan	750.63	1216.05	1397.39	700	761
Tongling	418.7	627.1	861.72	277	207
Anqing	829.95	1331.21	1497.64	1377	1497
Chizhou	246.28	420.96	550.31	471	415
Xuancheng	456.79	759.12	982.3	1145	987

es
2014-2016
8866
2707
5013
4244
10036
5072
3114
2753
2805
2864
5975
7392
5070
2759
4337
386
4059
3698
2438
2054
1133
430
1742
568
1410
