# Supplementary material for: The influencing factors of biomedical R&D cooperation in three major urban agglomerations of China based on cooperative patents
Source: PLoS One. 2023 Jan 4;18(1):e0278942. doi: 10.1371/journal.pone.0278942 (PMC9812333; doi:10.1371/journal.pone.0278942)
Supplement: S1 Data — (ZIP) [file pone.0278942.s001.zip › Original Files/Gross value of pharmaceutical manufacturing in urban agglomerations.pdf]

	Gross output value of pharmaceutical industry (100 million yuan)		
	2008-2010	2011-2013	2014-2016
Shanghai	347.41	519.43	654.9
Nanjing	94.76	189	261.16
Wuxi	90.39	166.69	218.74
Changzhou	82.02	122.71	159.53
Suzhou	152.97	231.9	286.27
Nantong	133.45	205.67	310.55
Yancheng	103.84	154.51	262.5
Yangzhou	46.81	82.07	116.24
Zhenjiang	13.34	25.72	41.92
Taizhou2	262.22	460.96	749.17
Hangzhou	158.87	246.03	371.16
Ningbo	29.52	48.72	66.67
Jiaxing	15.69	19.08	28.99
Huzhou	24.89	31.19	39.95
Shaoxing	149.08	212.21	280.54
Zhoushan	5.2	6.16	4.19
Jinhua	83.42	103.35	129.78
Taizhou1	192.38	248.38	326.13
Hefei	243.29	404.05	505.81
Wuhu	12.48	41.11	52.61
Maanshan	9.56	6.25	3.15
Tongling	2.41	3.44	3.7
Anqing	33.54	54.24	79.01
Chizhou	3.22	10.22	13.6
Xuancheng	5.5	13.11	23.75
