# Supplementary material for: The influencing factors of biomedical R&D cooperation in three major urban agglomerations of China based on cooperative patents
Source: PLoS One. 2023 Jan 4;18(1):e0278942. doi: 10.1371/journal.pone.0278942 (PMC9812333; doi:10.1371/journal.pone.0278942)
Supplement: S1 Data — (ZIP) [file pone.0278942.s001.zip › Original Files/Latitude and longitude table of city.pdf]

	longitud	latitude
Beijing	116.28	39.54
Tianjin	117.12	39.02
Shijiazh	114.30	38.02
Tangshan	118.11	39.36
Qinhuang	119.35	39.55
Handan	114.28	36.36
Xingtai	114.30	37.04
Baoding	115.30	38.51
Zhangjia	114.53	40.48
Chengde	117.57	40.59
Cangzhou	116.52	38.18
Langfang	116.42	39.31
