# Supplementary material for: The influencing factors of biomedical R&D cooperation in three major urban agglomerations of China based on cooperative patents
Source: PLoS One. 2023 Jan 4;18(1):e0278942. doi: 10.1371/journal.pone.0278942 (PMC9812333; doi:10.1371/journal.pone.0278942)
Supplement: S1 Data — (ZIP) [file pone.0278942.s001.zip › Original Files/GDP and number of companies in urban agglomeration.pdf]

	GDP (100 million yuan)			Number of companies		
	2008-2010	2011-2013	2014-2016	2008-2010	2011-20	2014-2016
Shanghai	15427.43	20398.52	25623.27	17794	9838	8866
Nanjing	4391.84	7142.43	9681.51	3210	2628	2707
Wuxi	5081.88	7406.18	8644.53	6925	5296	5013
Changzhou	2610.38	4000.29	5316.29	5940	3917	4244
Suzhou	8015.73	11899.55	14580.02	12405	10374	10036
Nantong	2977.2	4596.3	6189.76	6940	4986	5072
Yancheng	1979.35	3127.29	4208.07	3433	2775	3114
Yangzhou	1910.59	2961.17	4054.71	3551	2630	2753
Zhenjiang	1717.18	2638.98	3529.59	2897	2466	2805
Taizhou2	1718.64	2729.75	3720.19	3475	2550	2864
Hangzhou	5276.33	7721.53	10190.03	10103	6026	5975
Ningbo	4447.21	6590.11	8100.13	12216	6862	7392
Jiaxing	2011.18	2905.11	3577.51	6952	4339	5070
Huzhou	1149.37	1662.5	2108.21	3333	2438	2759
Shaoxing	2463.54	3651.11	4506.96	5341	3729	4337
Zhoushan	555.94	852.26	1116.44	615	385	386
Jinhua	1852.61	2709.21	3431.83	5471	3362	4059
Taizhou1	2139.06	2953.17	3562.79	6593	3383	3698
Hefei	2242.86	4161.65	5705.07	1837	2158	2438
Wuhu	993.04	1877.63	2488.77	1546	1765	2054
Maanshan	750.63	1216.05	1397.39	700	761	1133
Tongling	418.7	627.1	861.72	277	207	430
Anqing	829.95	1331.21	1497.64	1377	1497	1742
Chizhou	246.28	420.96	550.31	471	415	568
Xuancheng	456.79	759.12	982.3	1145	987	1410
