# Supplementary figures and images for: The influencing factors of biomedical R&D cooperation in three major urban agglomerations of China based on cooperative patents
Source: PLoS One. 2023 Jan 4;18(1):e0278942. doi: 10.1371/journal.pone.0278942 (PMC9812333; doi:10.1371/journal.pone.0278942)

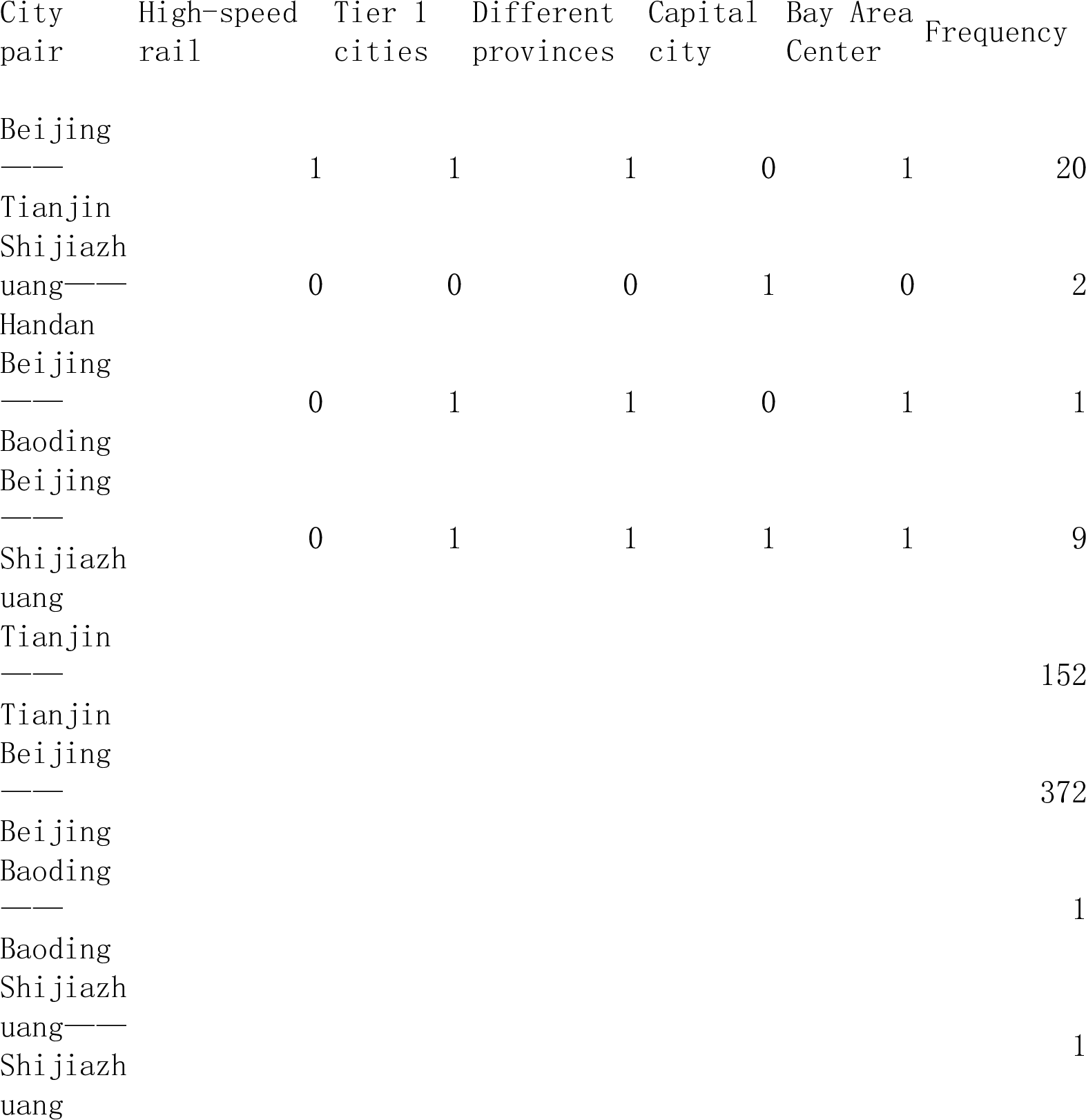

Supplement: S1 Data — (ZIP) [file pone.0278942.s001.zip › PACE Corrected/2008-2010Beijing-Tianjin-Hebei Urban Agglomeration.tif]

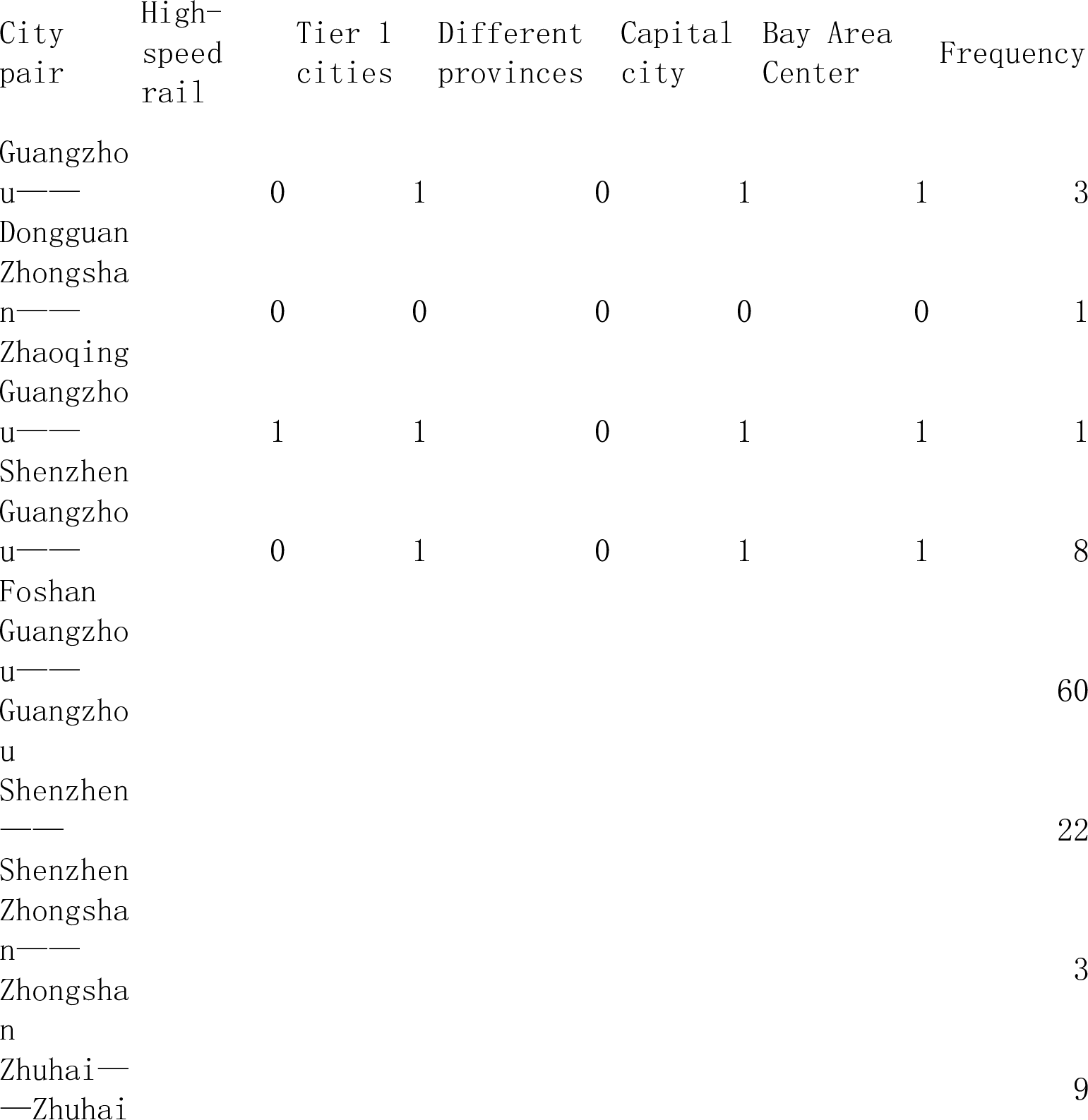

Supplement: S1 Data — (ZIP) [file pone.0278942.s001.zip › PACE Corrected/2008-2010the Pearl River Delta Urban Agglomeration.tif]

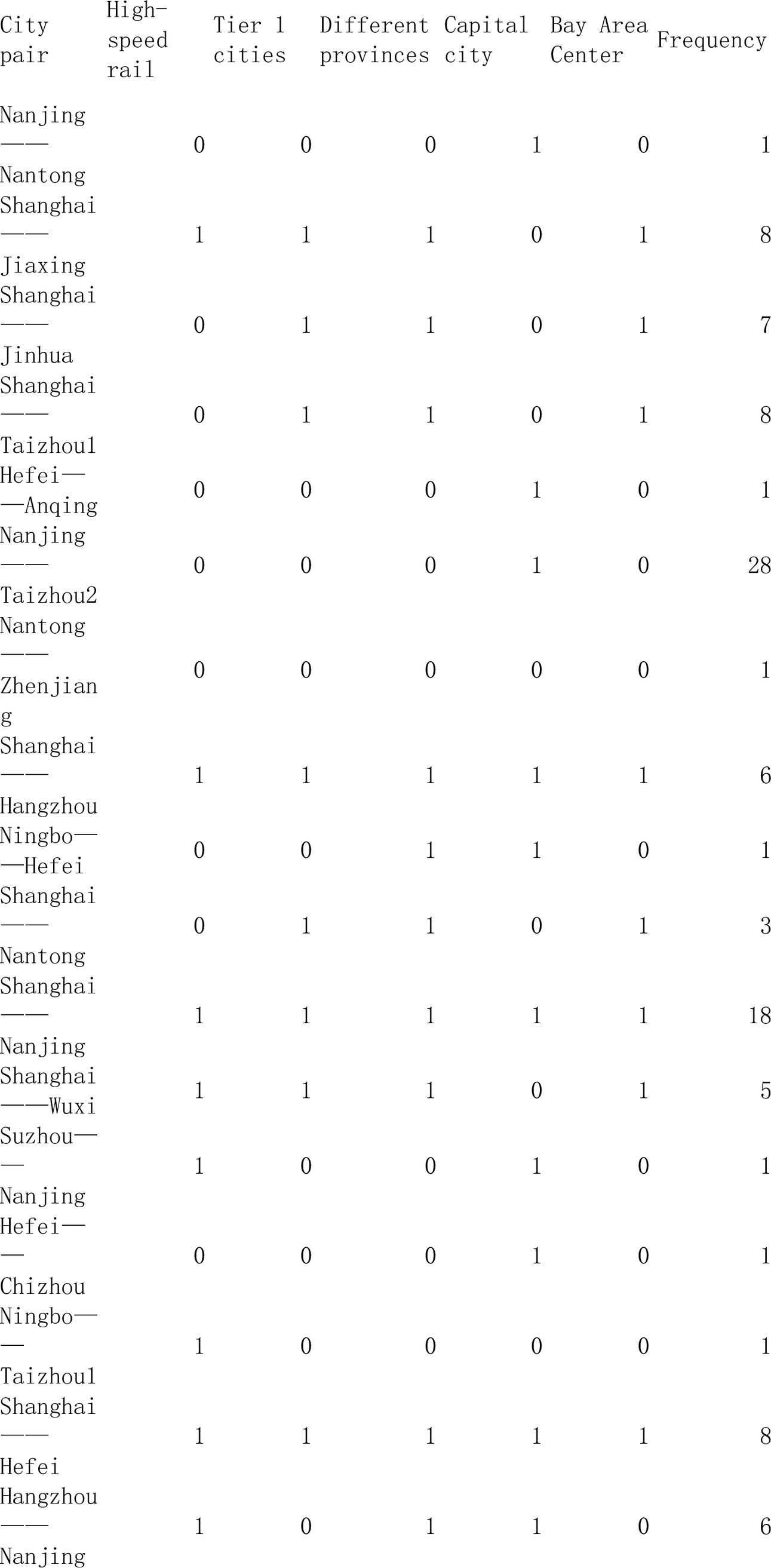

Supplement: S1 Data — (ZIP) [file pone.0278942.s001.zip › PACE Corrected/2008-2010Yangtze River Delta Urban Agglomeration.tif]

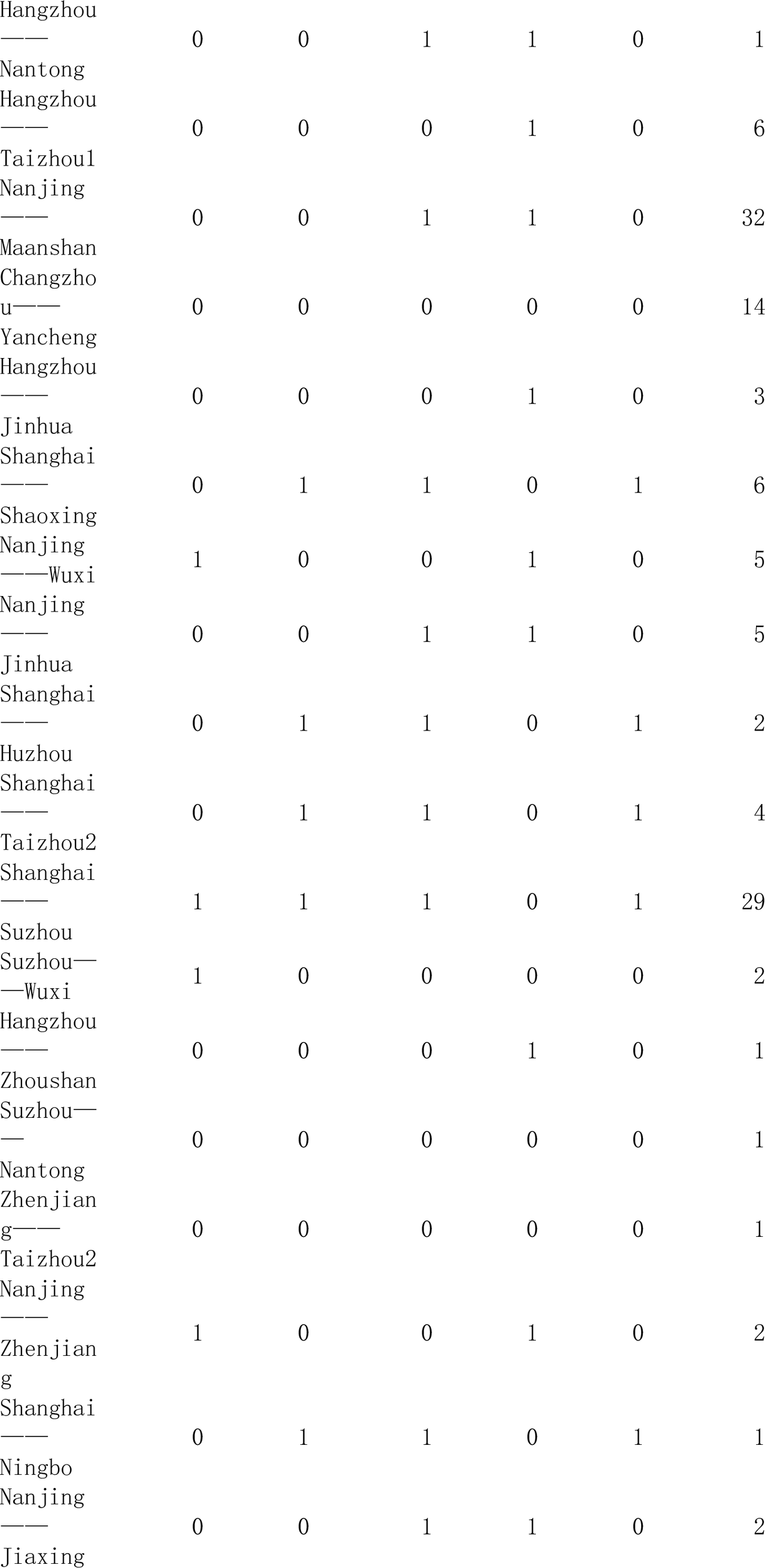

Supplement: S1 Data — (ZIP) [file pone.0278942.s001.zip › PACE Corrected/2008-2010Yangtze River Delta Urban Agglomeration.tif]

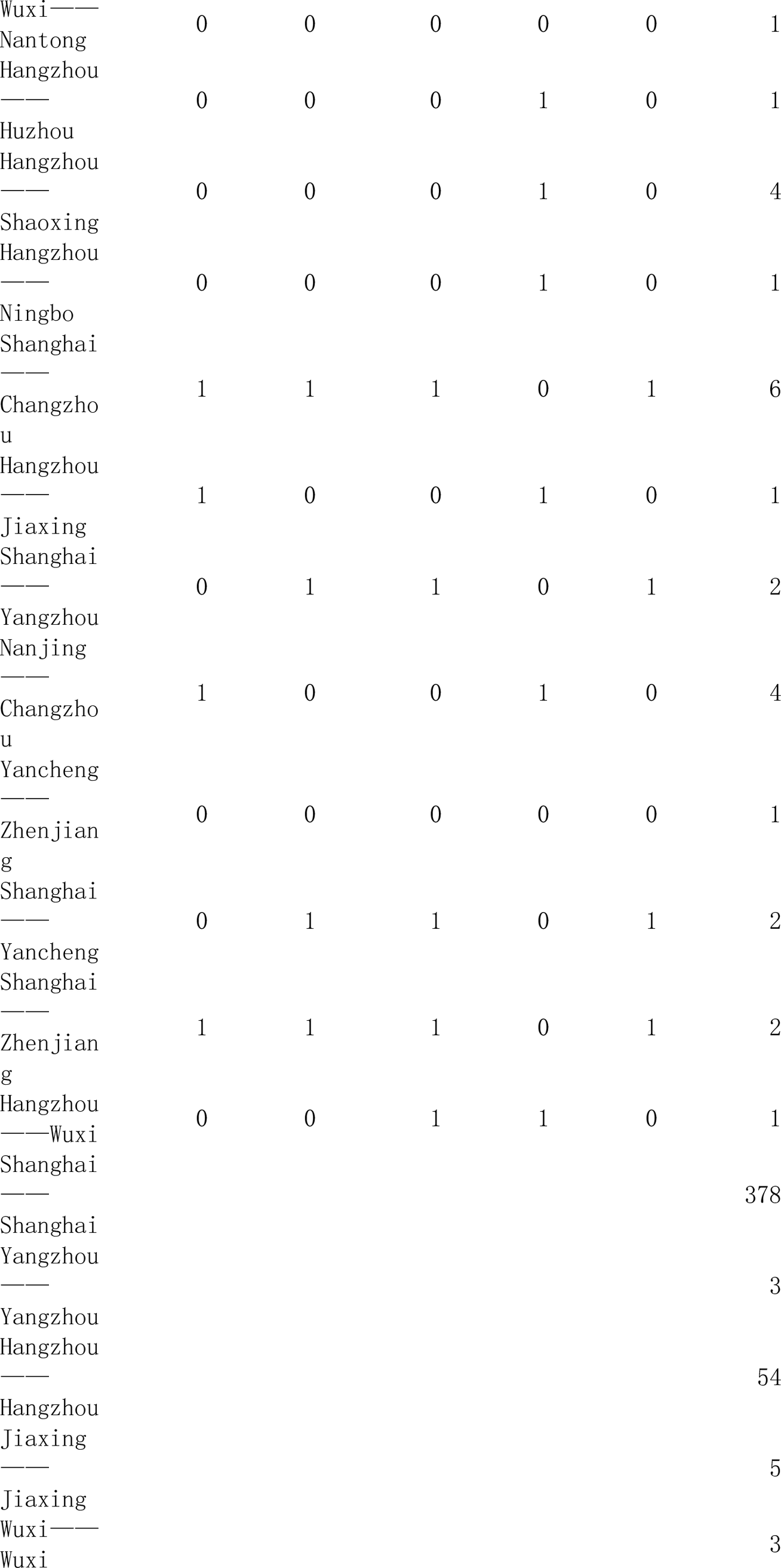

Supplement: S1 Data — (ZIP) [file pone.0278942.s001.zip › PACE Corrected/2008-2010Yangtze River Delta Urban Agglomeration.tif]

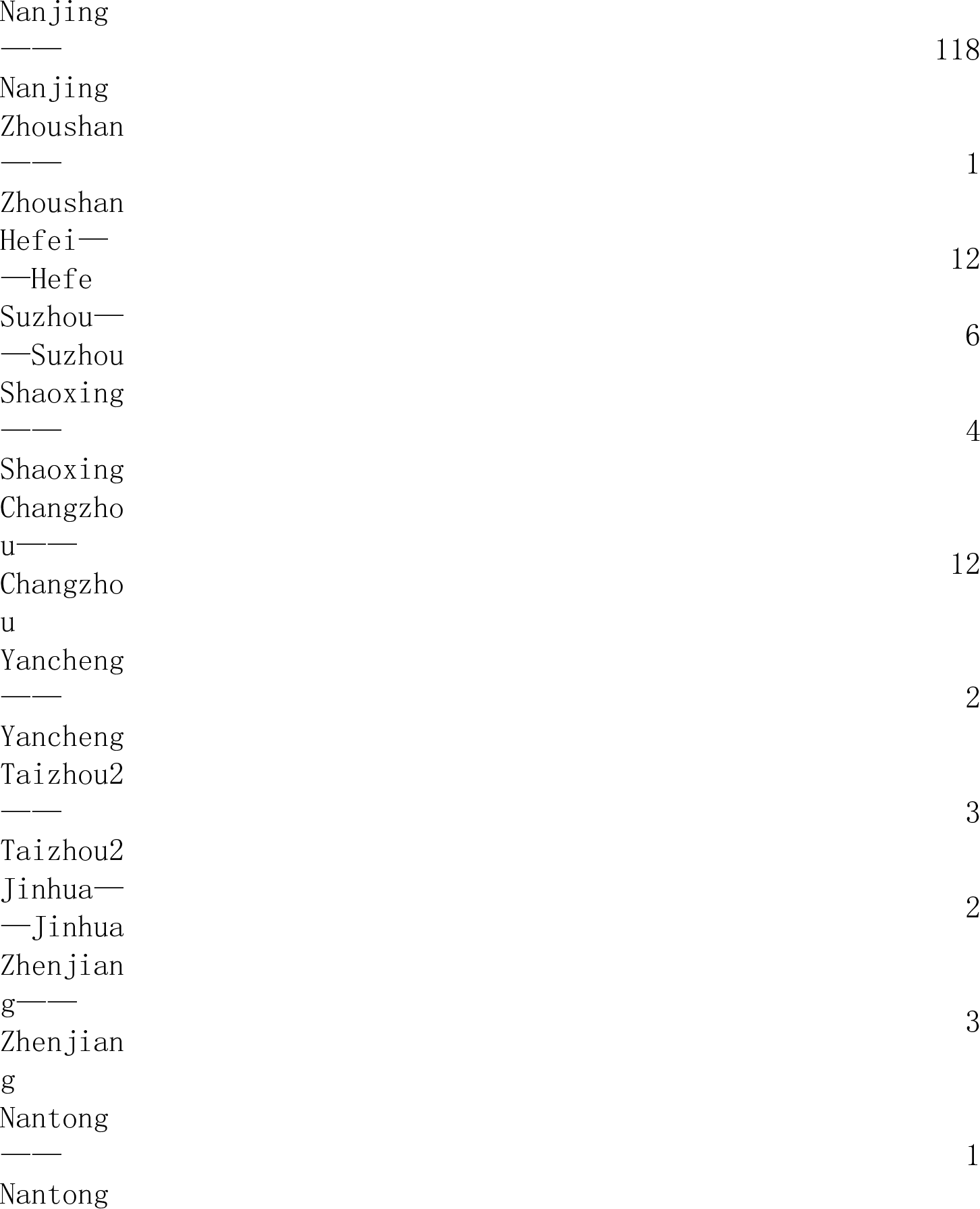

Supplement: S1 Data — (ZIP) [file pone.0278942.s001.zip › PACE Corrected/2008-2010Yangtze River Delta Urban Agglomeration.tif]

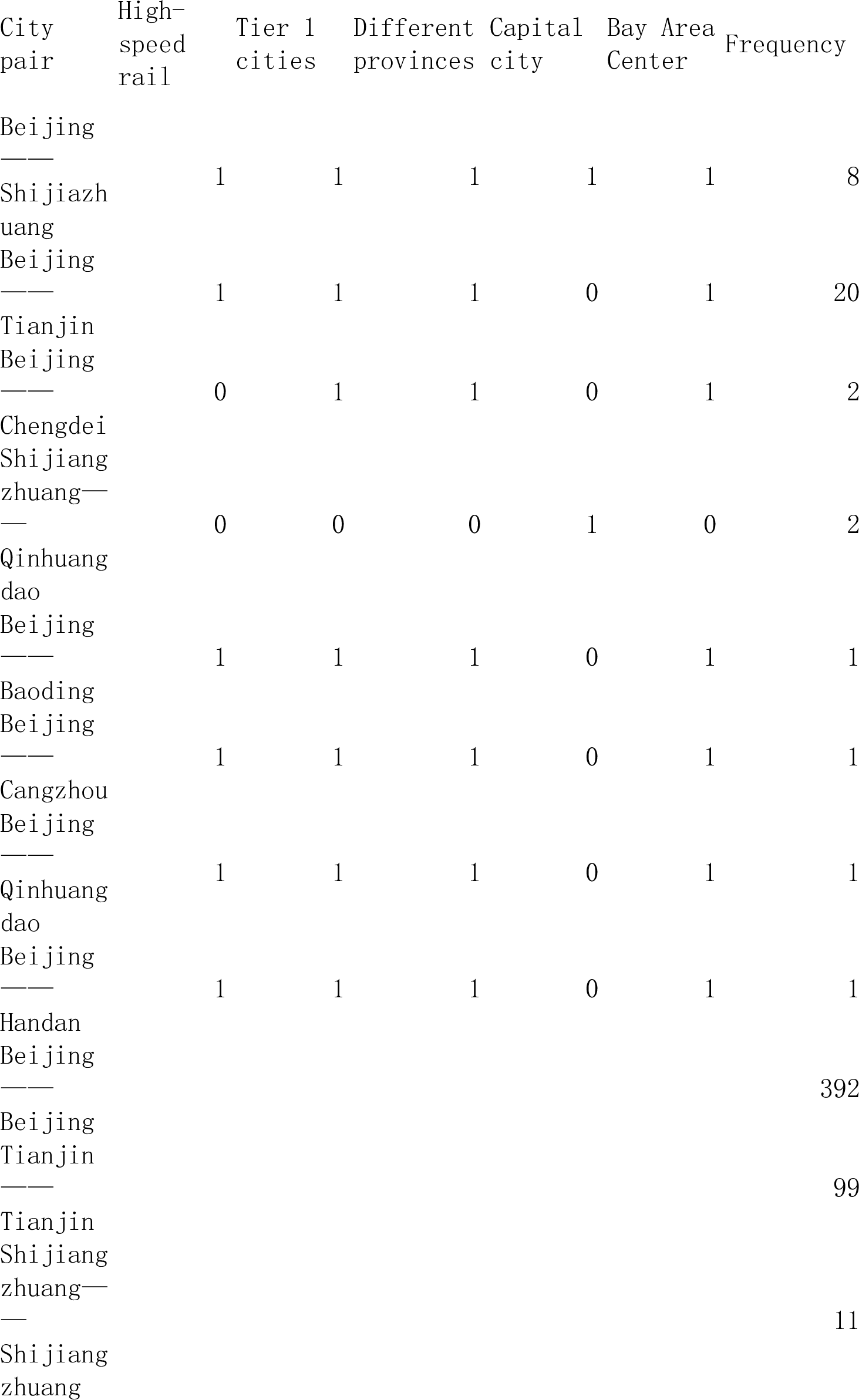

Supplement: S1 Data — (ZIP) [file pone.0278942.s001.zip › PACE Corrected/2011-2013Beijing-Tianjin-Hebei Urban Agglomeration.tif]

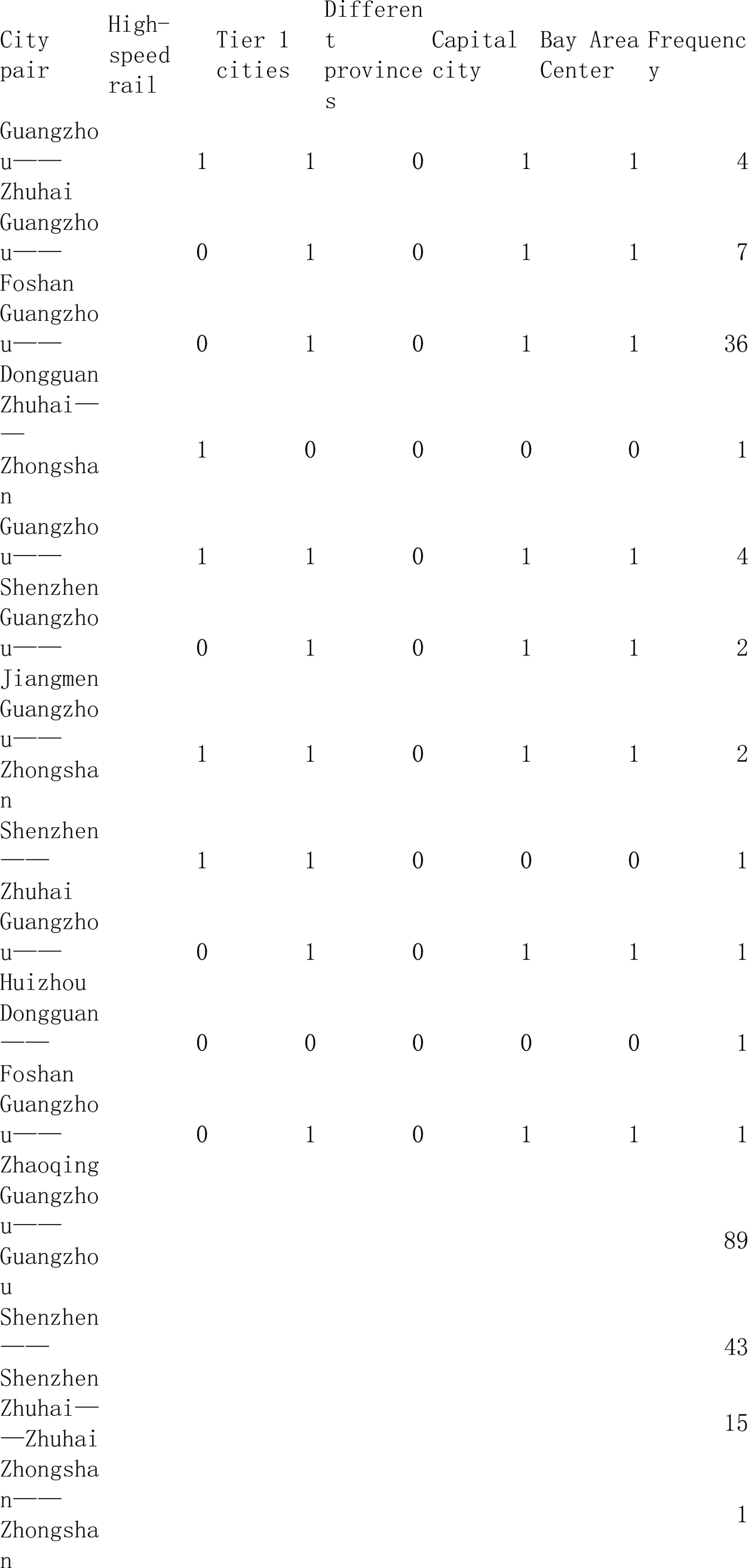

Supplement: S1 Data — (ZIP) [file pone.0278942.s001.zip › PACE Corrected/2011-2013the Pearl River Delta Urban Agglomeration.tif]

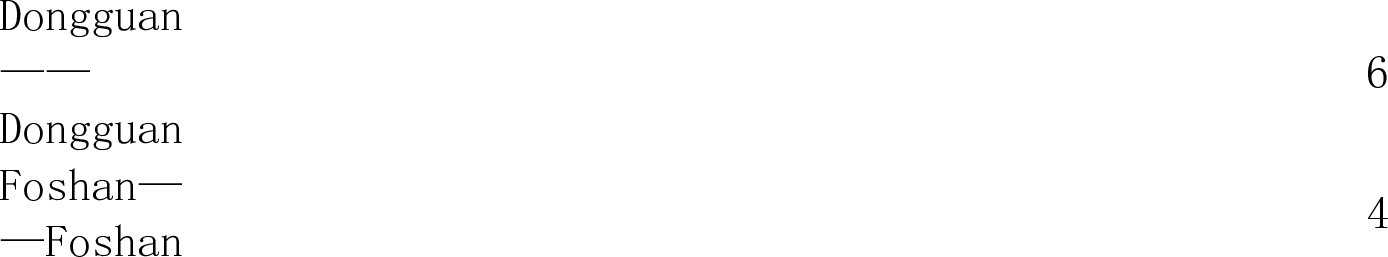

Supplement: S1 Data — (ZIP) [file pone.0278942.s001.zip › PACE Corrected/2011-2013the Pearl River Delta Urban Agglomeration.tif]

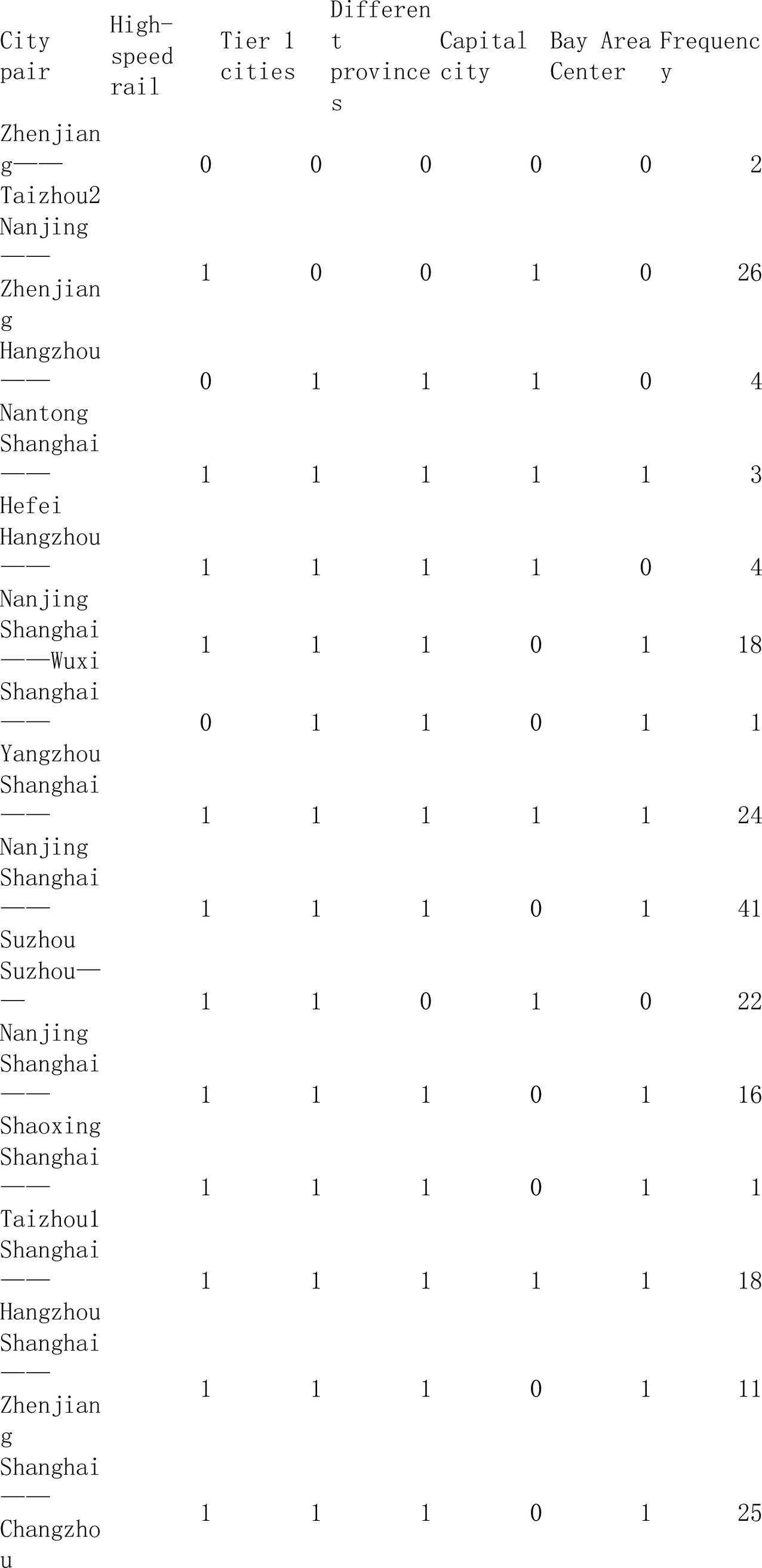

Supplement: S1 Data — (ZIP) [file pone.0278942.s001.zip › PACE Corrected/2011-2013Yangtze River Delta Urban Agglomeration.tif]

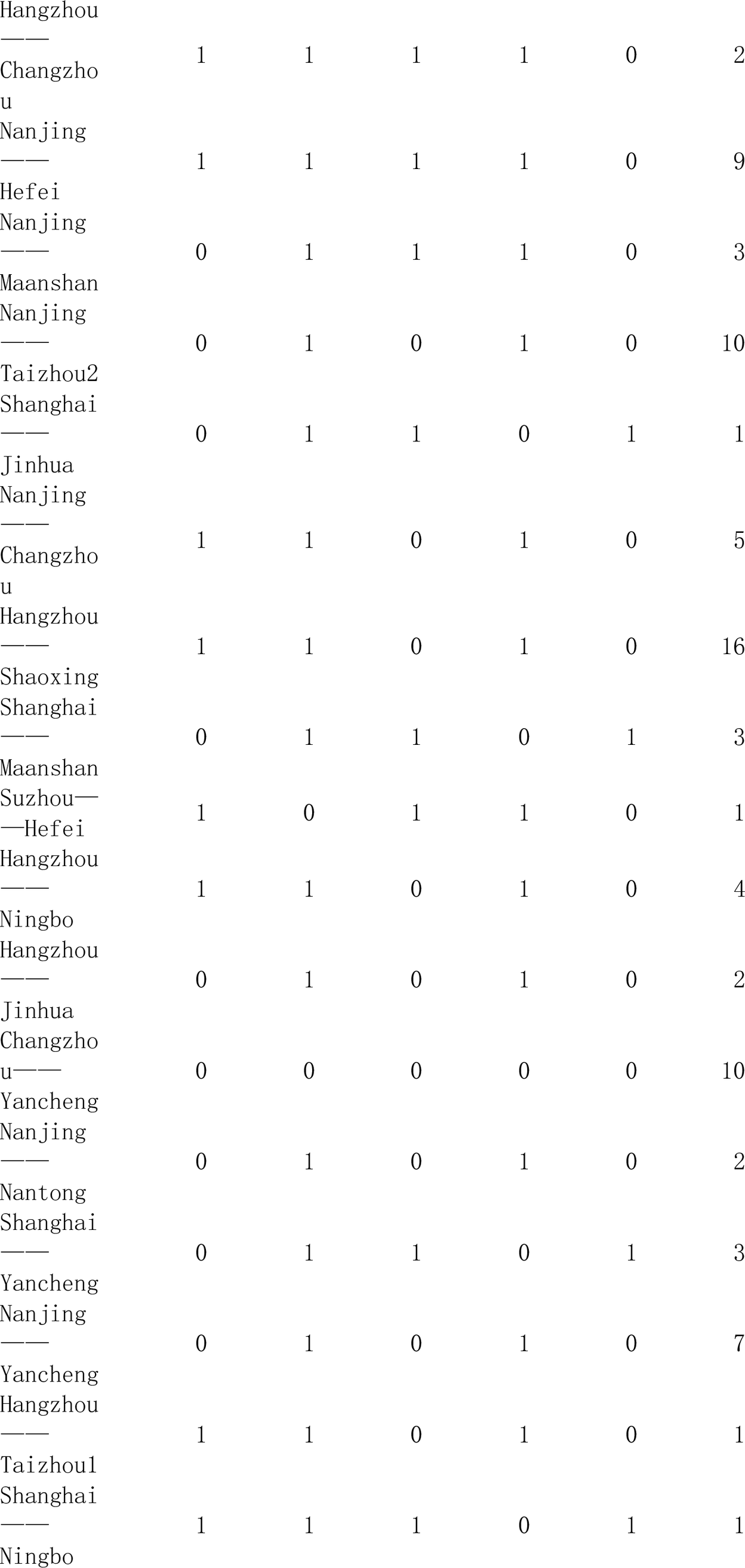

Supplement: S1 Data — (ZIP) [file pone.0278942.s001.zip › PACE Corrected/2011-2013Yangtze River Delta Urban Agglomeration.tif]

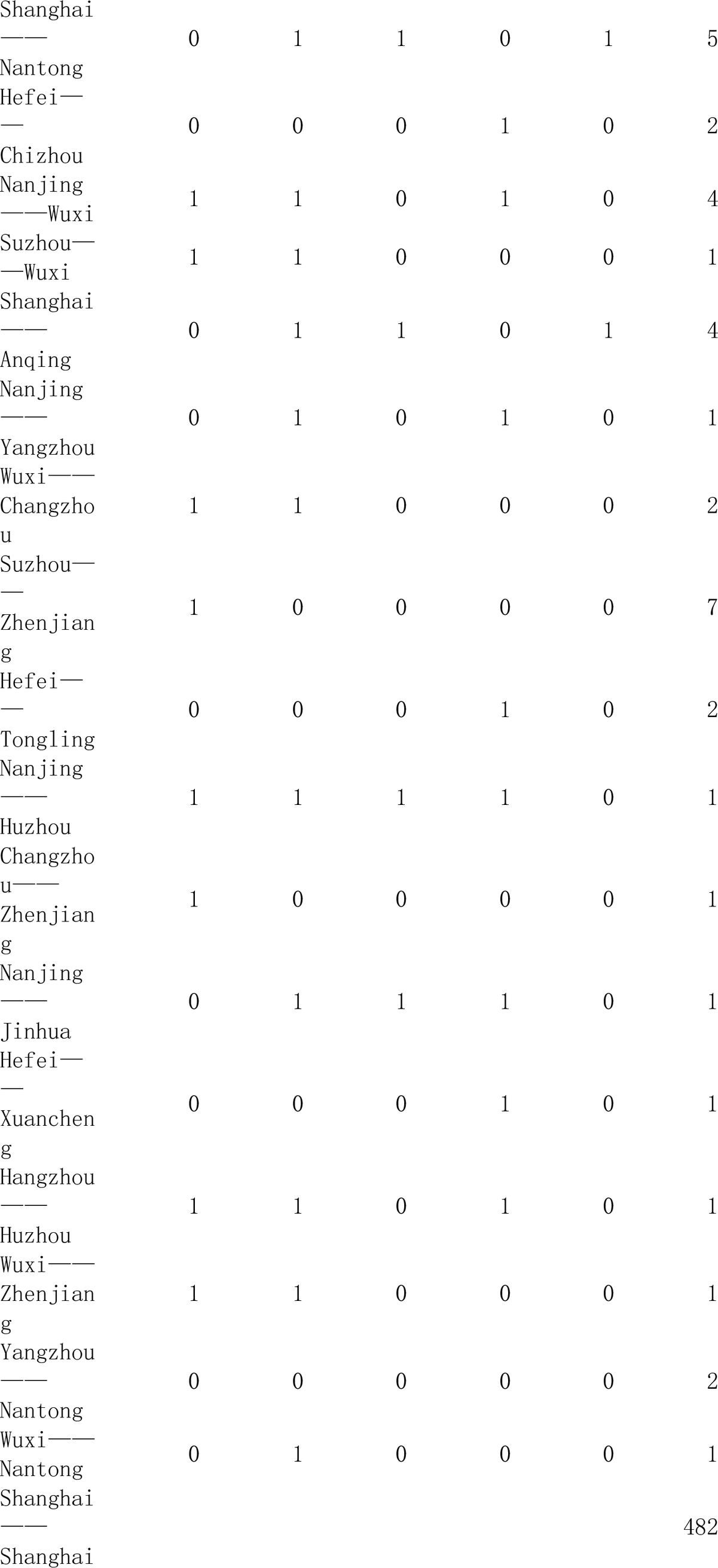

Supplement: S1 Data — (ZIP) [file pone.0278942.s001.zip › PACE Corrected/2011-2013Yangtze River Delta Urban Agglomeration.tif]

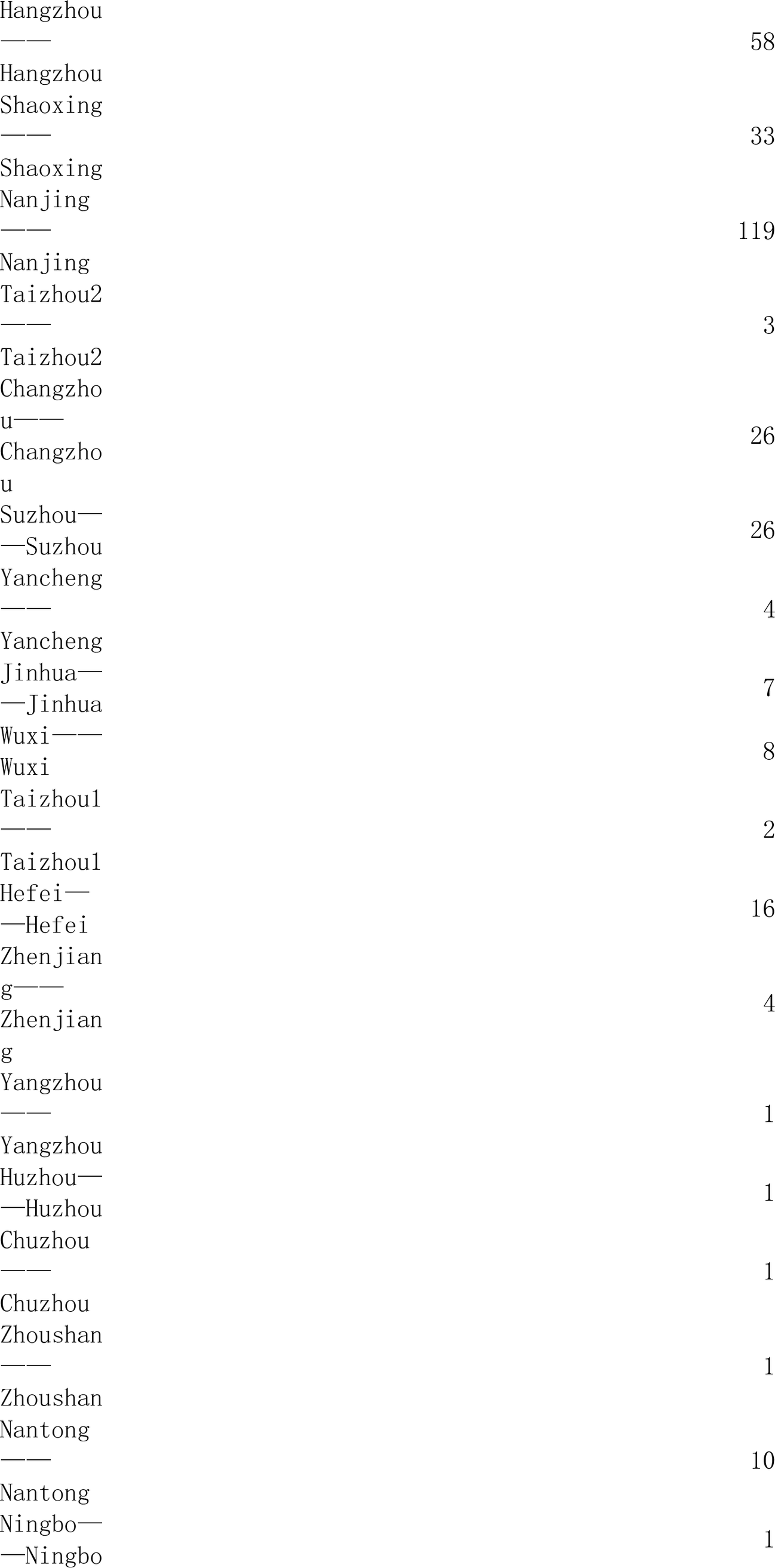

Supplement: S1 Data — (ZIP) [file pone.0278942.s001.zip › PACE Corrected/2011-2013Yangtze River Delta Urban Agglomeration.tif]

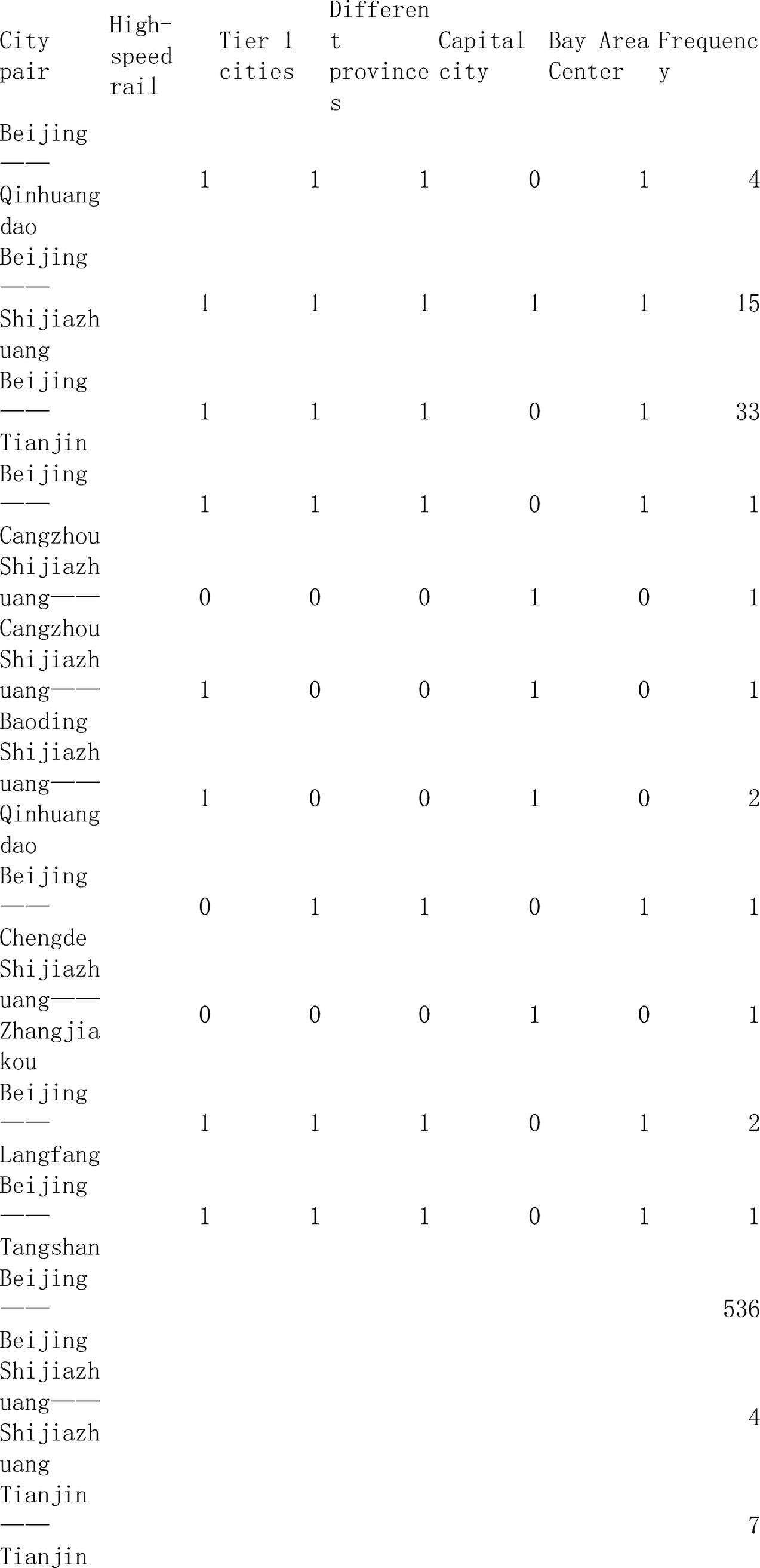

Supplement: S1 Data — (ZIP) [file pone.0278942.s001.zip › PACE Corrected/2014-2016Beijing-Tianjin-Hebei Urban Agglomeration.tif]

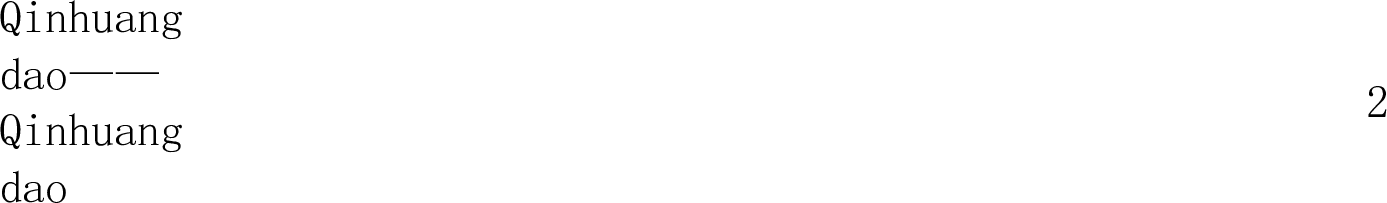

Supplement: S1 Data — (ZIP) [file pone.0278942.s001.zip › PACE Corrected/2014-2016Beijing-Tianjin-Hebei Urban Agglomeration.tif]

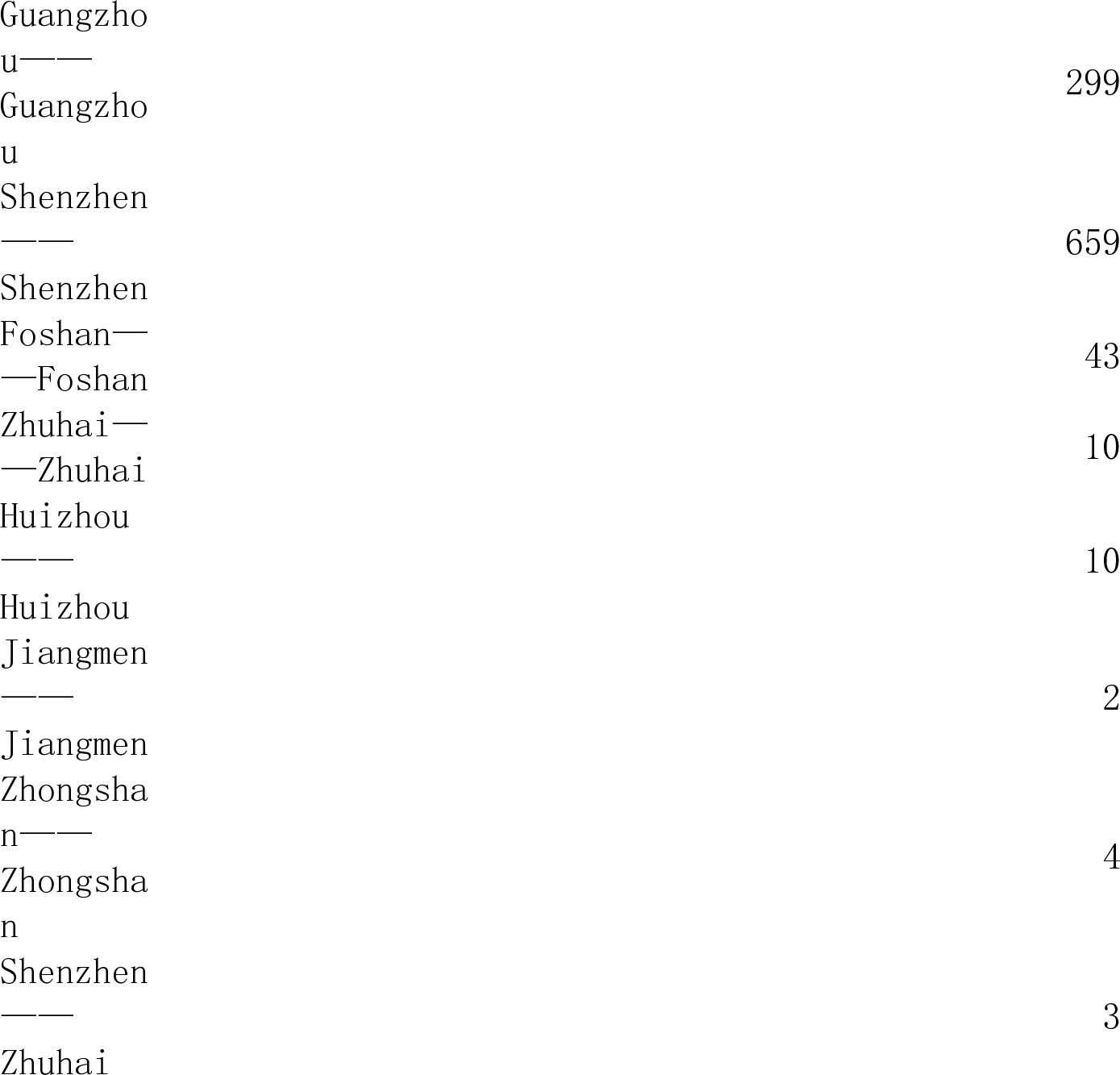

Supplement: S1 Data — (ZIP) [file pone.0278942.s001.zip › PACE Corrected/2014-2016the Pearl River Delta Urban Agglomeration.tif]

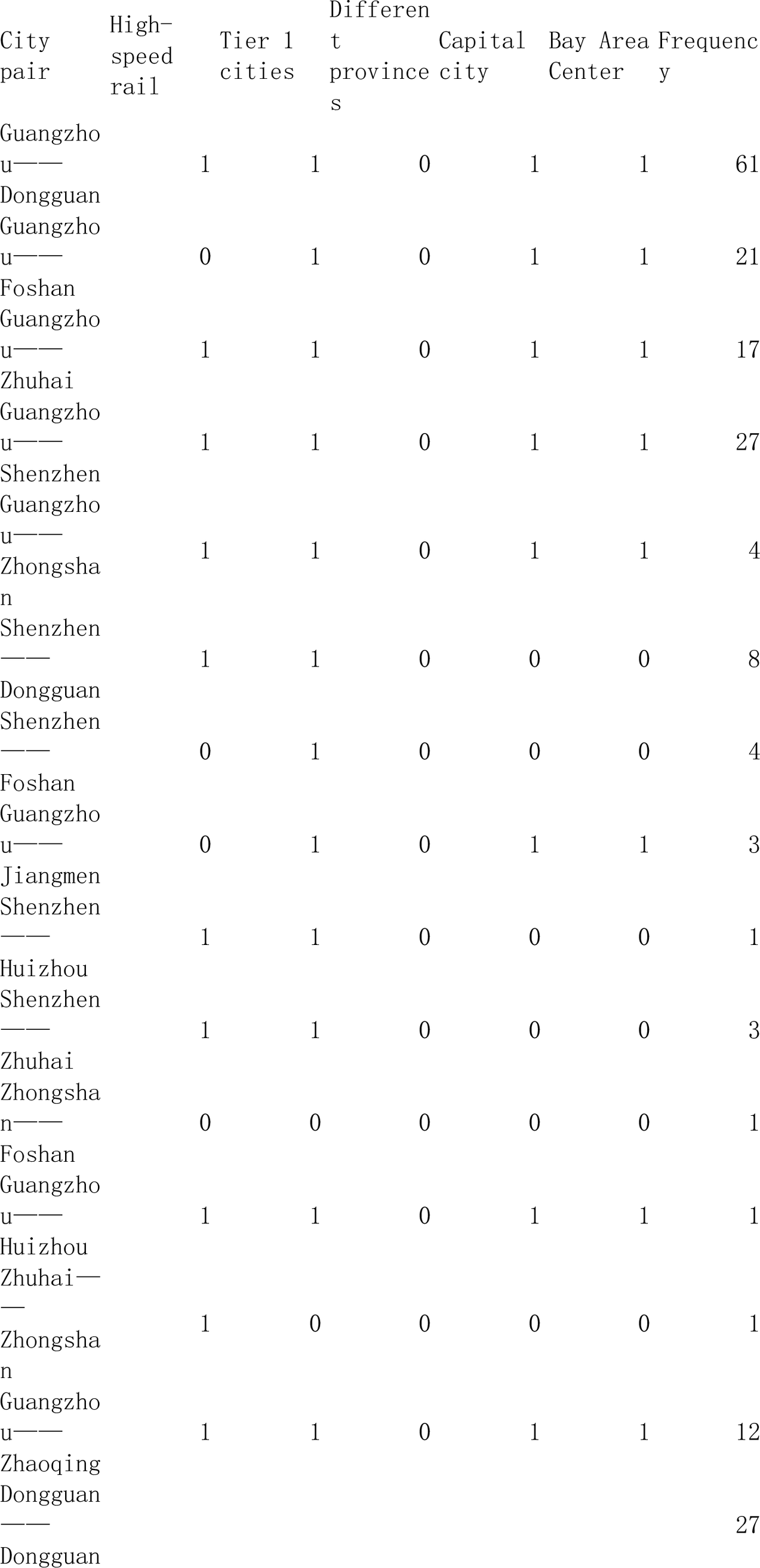

Supplement: S1 Data — (ZIP) [file pone.0278942.s001.zip › PACE Corrected/2014-2016the Pearl River Delta Urban Agglomeration.tif]

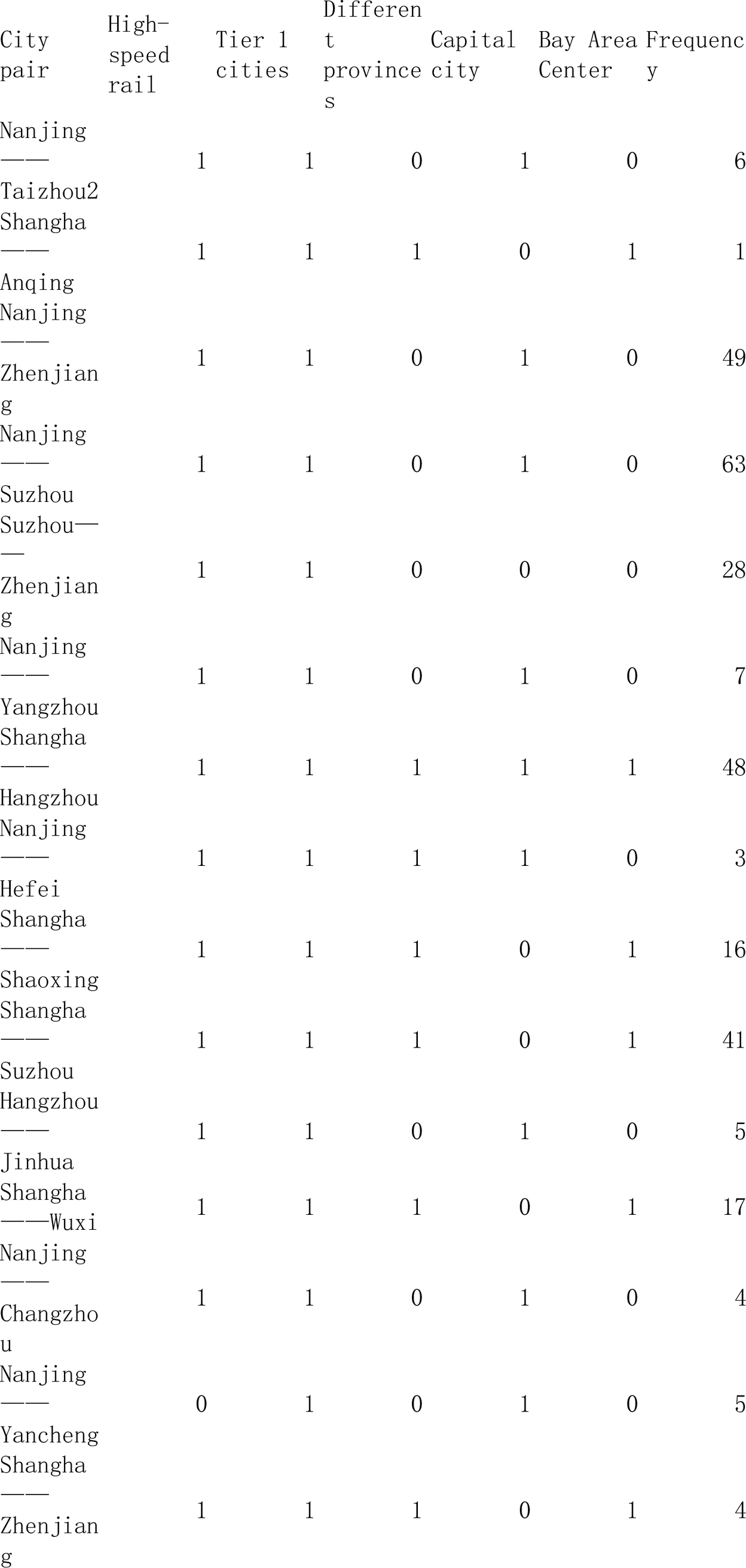

Supplement: S1 Data — (ZIP) [file pone.0278942.s001.zip › PACE Corrected/2014-2016Yangtze River Delta Urban Agglomeration.tif]

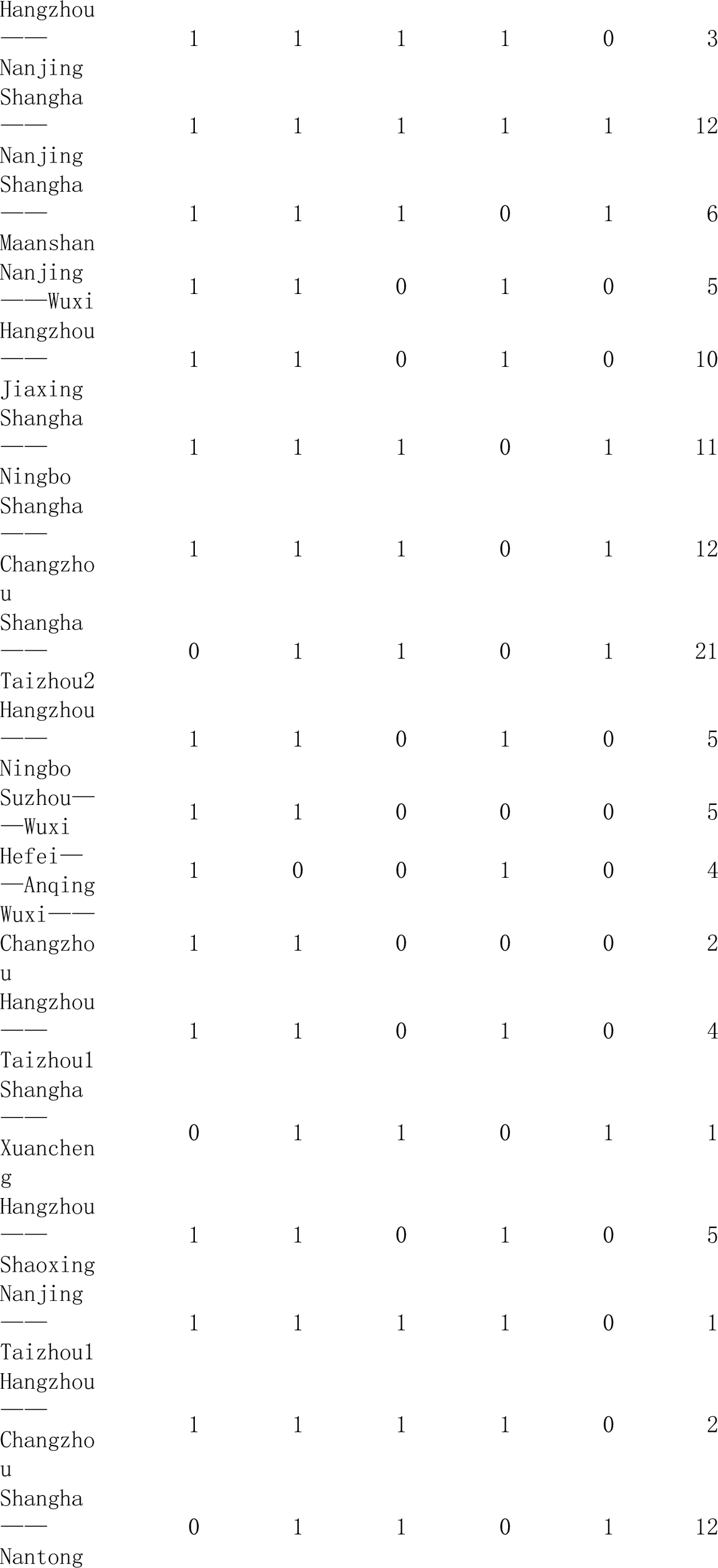

Supplement: S1 Data — (ZIP) [file pone.0278942.s001.zip › PACE Corrected/2014-2016Yangtze River Delta Urban Agglomeration.tif]

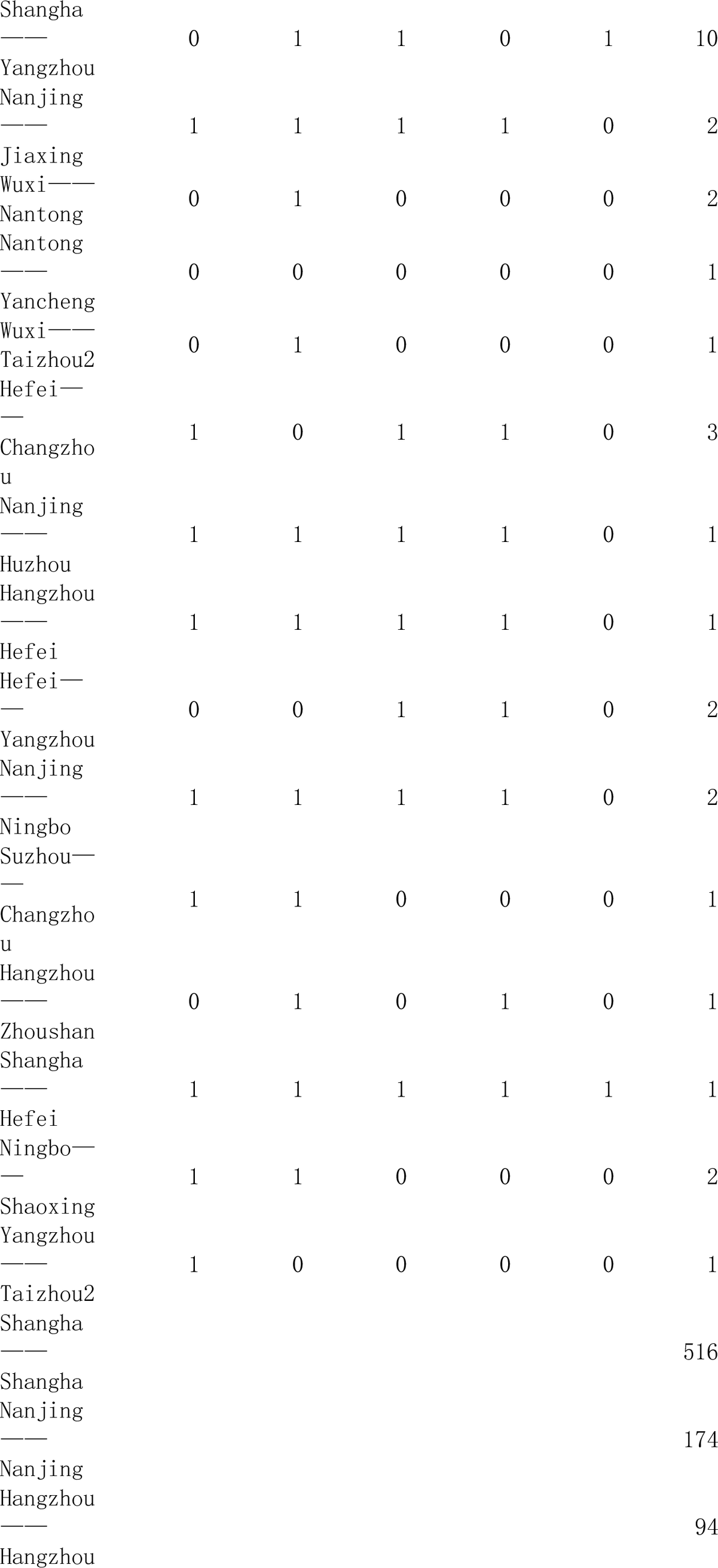

Supplement: S1 Data — (ZIP) [file pone.0278942.s001.zip › PACE Corrected/2014-2016Yangtze River Delta Urban Agglomeration.tif]

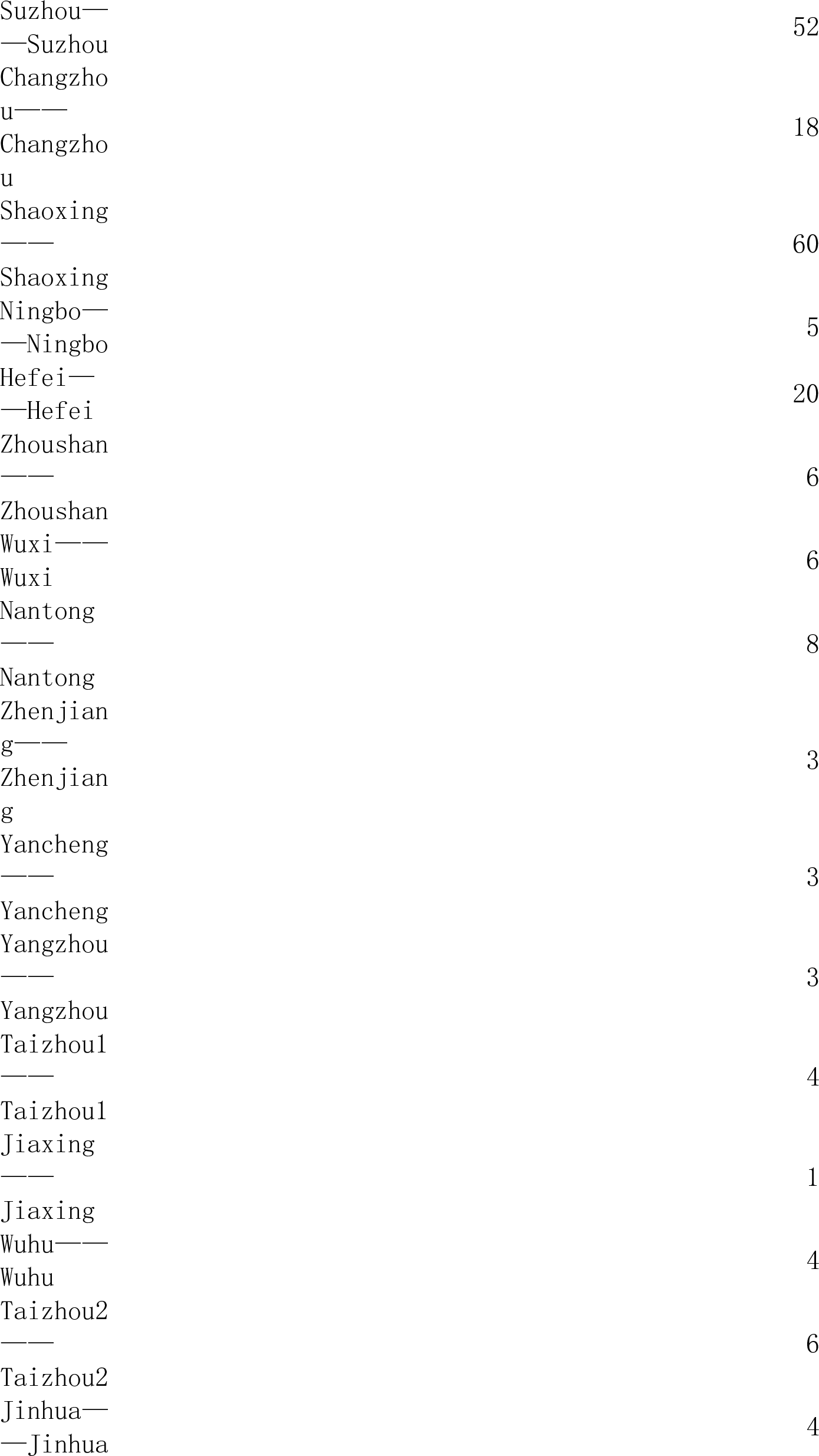

Supplement: S1 Data — (ZIP) [file pone.0278942.s001.zip › PACE Corrected/2014-2016Yangtze River Delta Urban Agglomeration.tif]

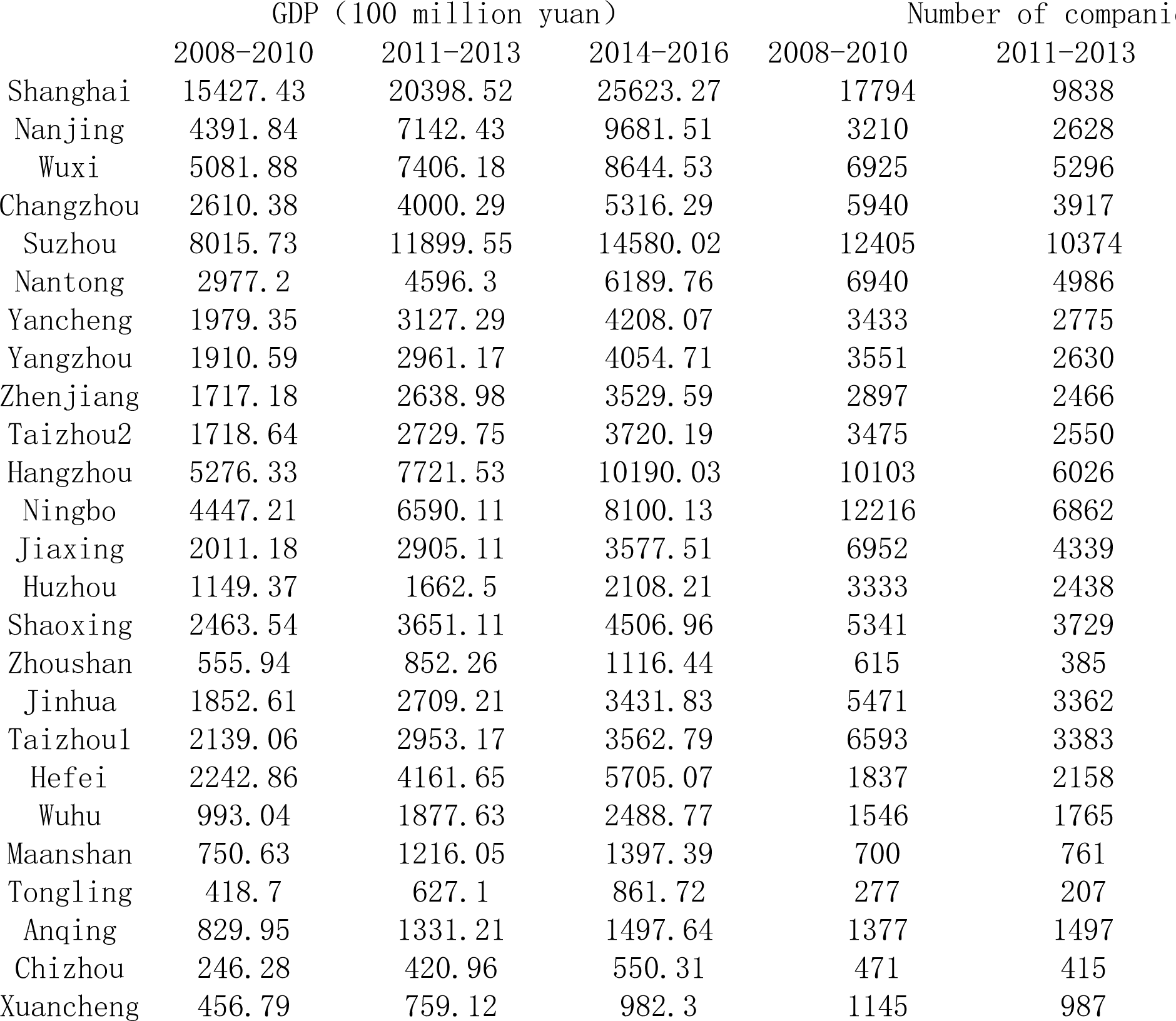

Supplement: S1 Data — (ZIP) [file pone.0278942.s001.zip › PACE Corrected/GDP and number of companies in urban agglomeration.tif]

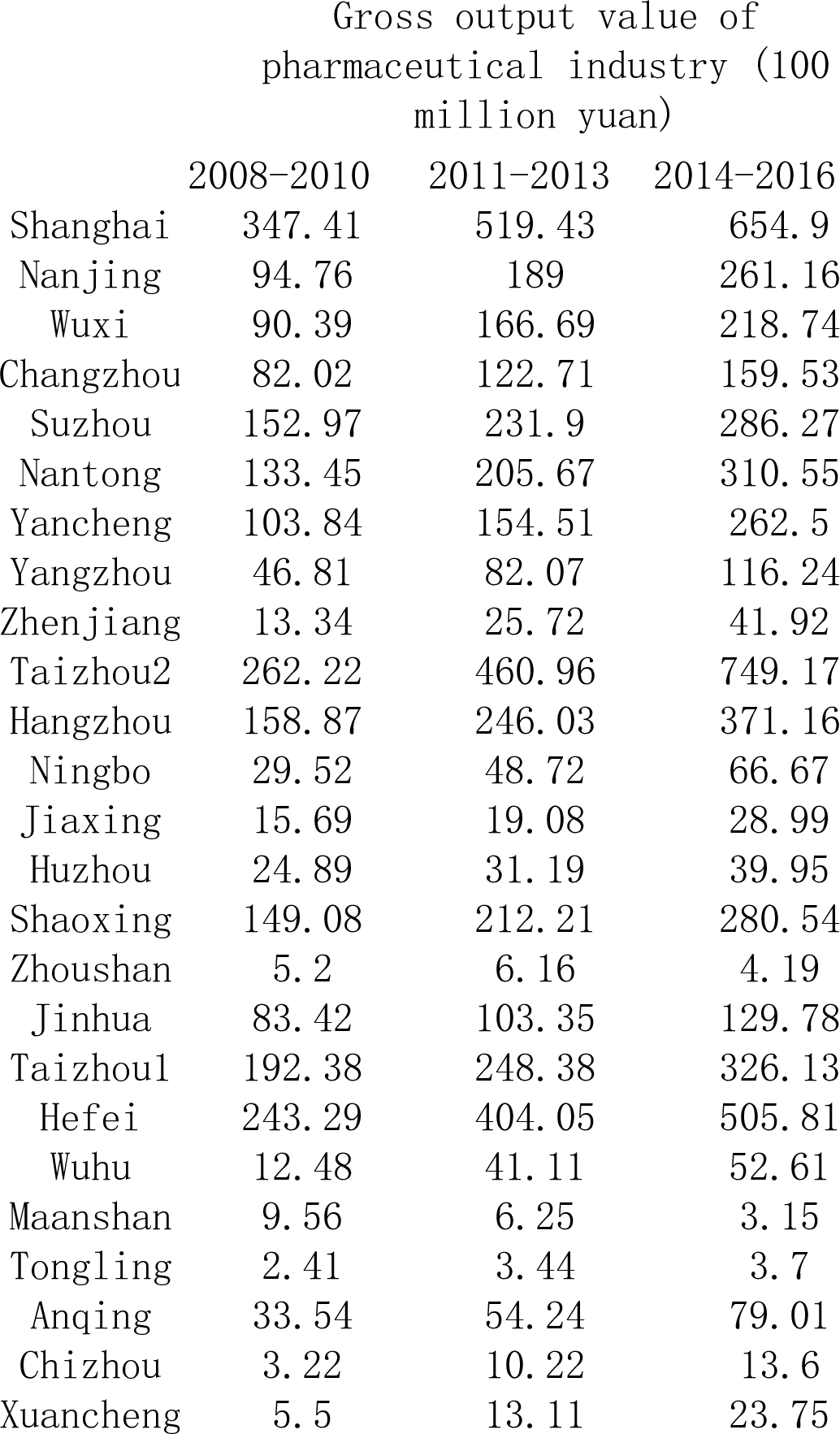

Supplement: S1 Data — (ZIP) [file pone.0278942.s001.zip › PACE Corrected/Gross value of pharmaceutical manufacturing in urban agglomerations.tif]

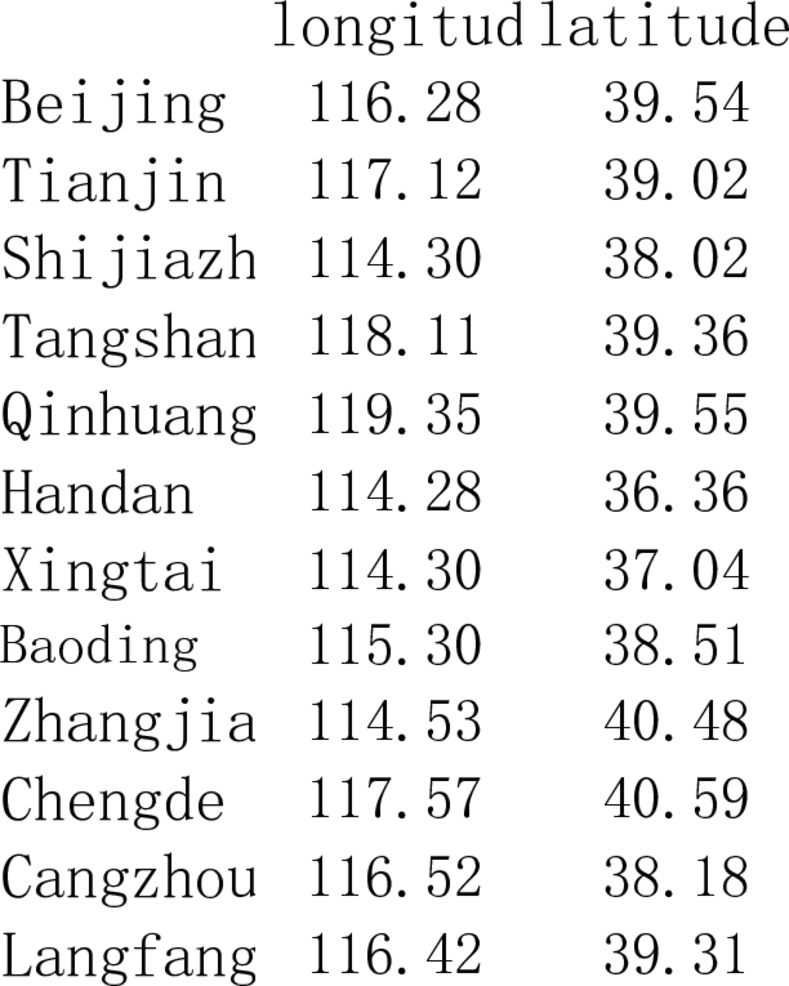

Supplement: S1 Data — (ZIP) [file pone.0278942.s001.zip › PACE Corrected/Latitude and longitude table of city.tif]

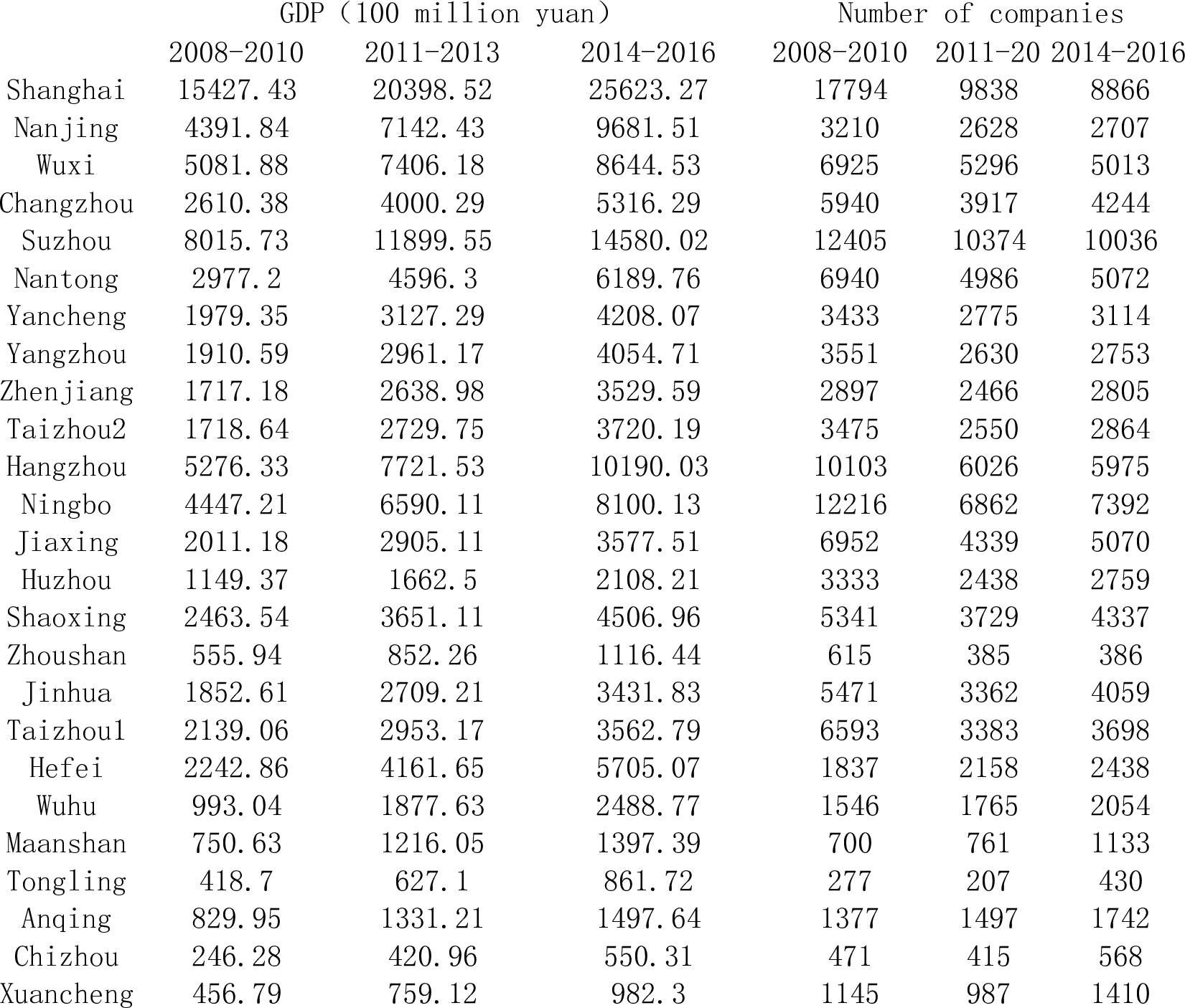

Supplement: S1 Data — (ZIP) [file pone.0278942.s001.zip › PACE Corrected/GDP and number of companies in urban agglomeration.tif]
