# Supplementary material for: The influencing factors of biomedical R&D cooperation in three major urban agglomerations of China based on cooperative patents
Source: PLoS One. 2023 Jan 4;18(1):e0278942. doi: 10.1371/journal.pone.0278942 (PMC9812333; doi:10.1371/journal.pone.0278942)
Supplement: Attachment — Submitted filename: rebuttal letter.docx [file pone.0278942.s002.docx]

**Rebuttal letter**

Dear reviewer：

On behalf of all authors，thank you very much for your suggestions to us. First of all, I would like to reply to the question raised by the original reviewer: “we suggest that you completely copy your manuscript in terms of language use, spelling and grammar.” I have used a software-Grammarly to modify it; Last returned due to failure to upload ● name of colleague or details of professional service organization editing your manuscript ● a copy of manuscript, showing your changes by highlighting or using track change ● (upload as * Support Information * file) ● clean copy of edited manuscript (upload as new * manuscript * file). The above problems have been solved one by one this time. Second, for your suggestions to me this time, I have uploaded the tracking change version of the manuscript file.

Yours sincerely.

Zuo-jun Dong
