# Supplementary material for: The influencing factors of biomedical R&D cooperation in three major urban agglomerations of China based on cooperative patents
Source: PLoS One. 2023 Jan 4;18(1):e0278942. doi: 10.1371/journal.pone.0278942 (PMC9812333; doi:10.1371/journal.pone.0278942)
Supplement: Attachment — Submitted filename: rebuttal letter.docx [file pone.0278942.s003.docx]

**Rebuttal letter**

Dear reviewer：

On behalf of all authors，thank you very much for your suggestions to us.

First of all,, for review 1 suggestion: 1 in chapter 1.1, we update the literature; 2. The proposed increase in chapter 2.1.1 biomedical research in the impacting factors of r&d cooperation in literature. This chapter mainly eliminate the standard of literature is not a tautology research literature, study design in chapter 2.2, have expounded the literature; 3. Select cities in chapter 2.3.1 is mainly based on the population base (> 21.89 million) of a standard; 4. In chapter 2.3.2, divided into periods according to the annual statistics too little, for the convenience of the statistics, chose a statistics every 2 years; 5. Increase the figure of 3.4 spatial structure evolution, more research and development of biomedical image analysis spatial pattern characteristics of cooperation.

Then, for advice from the reviewer 2:1. The part of the basic problems, such as grammar changes; 2. The conclusion part, will discuss with repetitive parts cut.

Yours sincerely.

Zuo-jun Dong
